# An update on the underlying risk factors of eating disorders onset during adolescence: a systematic review

**DOI:** 10.3389/fpsyg.2023.1221679

**Published:** 2023-11-08

**Authors:** Carmen Varela, Ángela Hoyo, María Eugenia Tapia-Sanz, Ana Isabel Jiménez-González, Benito Javier Moral, Paula Rodríguez-Fernández, Yadirnaci Vargas-Hernández, Luis Jorge Ruiz-Sánchez

**Affiliations:** ^1^Universitat de Barcelona, Barcelona, Spain; ^2^Departamento de Ciencias de la Salud, Facultad de Ciencias de la Salud, Universidad de Burgos, Burgos, Spain; ^3^Área de Psicología, Facultad de Ciencias de la Salud, Universidad Isabel I, Burgos, Spain; ^4^Facultad de Humanidades y Ciencias Sociales, Universidad Isabel I, Burgos, Spain

**Keywords:** eating disorders, adolescence, prevention, risk factors, protective factors

## Abstract

**Introduction:**

Eating disorders (EDs) are serious psychological problems that affect not only the individual, but also their entire environment. The prevalence rates of EDs are higher among the adolescent population. A better understanding of ED risk factors is essential to design effective prevention and intervention programs that focus beyond the areas of weight and appearance.

**Methods:**

The main objective of this systematic review was to identify the risk factors of EDs and provide a comprehensive approach, analyzing the interplay between individuals, their inner circle, and the society characteristics. The Web of Science, Scopus, CENTRAL and PsycInfo databases were searched.

**Results:**

The initial search produced 8,178 references. After removing duplicates and performing the selection process by three independent reviewers, 42 articles were included in the systematic review according to the pre-specified inclusion criteria. The results suggest the relevance of society and the inner circle on the development of EDs.

**Discussion:**

The internalization of the thin ideal, promoted by the current society, and living in an unsupportive, unaffectionate, non-cohesive environment were associated with the onset of EDs symptomatology. Other associated variables with this ED indicator were poor-quality relationships and feeling judged about appearance. These aspects seem to be essential for the development of individual characteristics like self-esteem or adaptative coping during adolescence. This systematic review has shown the complex etiology of EDs and the relevance of the interplay between the different areas involved. Furthermore, this information could be relevant to improve the design of innovative and more effective prevention and intervention programs.

**Systematic review registration:**

PROSPERO, identifier CRD42022320881.

## 1. Introduction

Eating disorders (EDs) are psychological conditions characterized by specific and severe disturbances in eating behavior, resulting from distressing thoughts and emotions mainly related to weight, body shape. However, some EDs like avoidant/restrictive food intake disorder (ARFID) or Binge Eating Disorder (BED) are more focused on aspects like food or intake (López and Treasure, 2002; Gaete and López, 2020). These are serious and potentially life-threatening disorders that can affect people’s emotional and physical health as well as their social functioning (O’Brien et al., 2017; Gaete and López, 2020). Types of eating disorders include anorexia nervosa (AN), bulimia nervosa (BN), BED, ARFID, and other specified feeding, and eating disorder (OSFED; American Psychiatry Association, 2013).

The prevalence of EDs is variable and complex due to changes in diagnostic criteria and differences between geographical regions. In the last decade, different reviews (Smink et al., 2012; López, 2017) have shown that the countries with the highest prevalence of EDs are Switzerland (12%), Chile (8.3%), and Spain (6.2%), followed by Colombia (4.5%), the United Kingdom (3.7%), and Portugal (3.1%; López, 2017). In countries such as the United States, EDs lifetime prevalence varies between 0.5 and 1.5% (Smink et al., 2012). Prevalence also varies between different age and gender groups, but a common feature is that EDs are more frequent in women than in men, in all countries and at all ages. Concretely, one study showed that the weighted means of lifetime EDs were 2.2% for men and 8.4% for women (Galmiche et al., 2019). Moreover, EDs usually begin in adolescence, a time when major psychological changes related to identity and physical appearance, as well as the development of self-regulation, occur (Smink et al., 2012; Galmiche et al., 2019; Stice et al., 2021).

Considering the above-described evidence, it is not surprising that the World Health Organization (WHO) has considered EDs as a priority problem among adolescents, given the health risk that these disorders imply, and the high rate of comorbidity with other types of disorders (Gibson et al., 2019; Stice et al., 2021). Some of the disorders that show the highest comorbidity in adolescents are the following: depression, anxiety, and obsessive-compulsive disorder (Gaete and López, 2020; Hambleton et al., 2022). They are also associated with personality disorders, substance abuse, and self-harming behaviors (Hambleton et al., 2022). Finally, there is also a high association between EDs and suicidal behavior in adolescents (Gibson et al., 2019). In this regard, during the pandemic a study showed that 65% of female adolescents with EDs had suicidal ideation and 45% had attempted suicide. Although the quarantine situation could increase suicide rates, there is a significant association between suicidal thoughts and behaviors and EDs (Semenova et al., 2022). This makes EDs one of the disorders that require further research in the field of prevention and intervention in adolescence, especially given that the current intervention programs have shown mixed efficacy (Pratt and Woolfenden, 2002; Swanson et al., 2011; Fairburn et al., 2015; Stice et al., 2021).

Understanding the risk factors involved in the onset of EDs is essential for the development of effective prevention and early intervention programs. Research has shown that a variety of risk factors may be involved, such as biological, psychological, familiar, and socio-cultural factors (Stice, 2016; Solmi et al., 2020; Barakat et al., 2023), with psychological-type factors being most associated with eating disorder symptomatology in adolescents (Suarez-Albor et al., 2022). Factors that have shown such an association include body dissatisfaction, (e.g., Fortes et al., 2013; Lazo et al., 2015; Gismero-González, 2020), social difficulties, poor to no support network (Cardi et al., 2018), tendency toward perfectionism (Pamies and Quiles, 2014; Laporta et al., 2020), impulsivity (Nuño-Gutiérrez et al., 2009), low self-esteem in relation to weight and image (Fairburn et al., 2003; Serpell and Troop, 2003), emotional dysregulation (Monell et al., 2018), and family environment (Cerniglia et al., 2017). Despite this evidence, there have been no systematic reviews published in recent years that have specifically identified the psychological risk factors that may predict the onset of EDs in adolescents. Stating the art of this question is essential to design prevention and intervention programs that effectively address the right psychological targets (Stice et al., 2021). The focus on psychological factors is due to improvements in psychological interventions and current eating disorder prevention programs. For that reason, biological and genetic factors have not been included in this systematic review, although their knowledge is relevant to provide a comprehensive approach. A multidisciplinary team must be involved in the development of future proposals Therefore, the aim of the present study is to conduct a systematic review to provide a comprehensive and updated view of the psychological risk factors that predict the onset of EDs in adolescents. This information could be useful to design innovative prevention and intervention programs for adolescent population, highlighting areas beyond weight and appearance.

## 2. Methods

A systematic review of the literature was conducted. The international prospective register for systematic reviews (PROSPERO) accepted the protocol of this systematic review on 3^rd^ June 2022, registration number CRD42022320881. This systematic review follows the guideline of Preferred Reporting Items for Systematic Reviews and Meta-Analyses (PRISMA) (Page et al., 2021).

### 2.1. Eligibility criteria

Adapted for a systematic review of association, the Population, Intervention, Comparator, Outcome and Study (PICOS) framework was used to establish the eligibility criteria of this study (Higgins and Green, 2008; Moola et al., 2015). As a result, the following inclusion criteria’s were: (a) population: adolescents between 11 and 19 years old; (b) exposure variables: psychological risk variables related to the onset of an ED (i.e., impulsivity, emotional dysregulation, social network exposure, perfectionism, self-demand, self-esteem, interpersonal relationships -social anxiety or social skills-, fear of maturing, low social or family support, and expressed emotion); (c) outcome: presence of ED symptomatology assessed by self-report or standardized/validated tools; and (d) type of study: observational studies that establish an association between exposure variables and outcome. Thus, adolescents who did not fall within the defined age range were excluded, as well as comorbidity with other serious physical or psychological problems, editorials, and conference abstracts. Table 1 shows the criteria for considering studies.

**Table 1:** Eligibility criteria to select studies for the systematic review.

Eligibility criteria	
Population	Adolescents between 11 and 19 years old
Exposure variables*	Psychological risk variables related to the onset of an ED (i.e., impulsivity, emotional dysregulation, social network exposure, perfectionism, self-demand, self-esteem, interpersonal relationships -social anxiety or social skills-, fear of maturing, low social or family support, and expressed emotion)
Outcome	Presence of ED symptomatology assessed by self-report or standardized/validated tools
Study	Observational studies that establish an association between exposure variables and outcome

* The PICOS framework is adapted for association systematic reviews. In this case there is no comparison (C), and the intervention (I) is replaced by Exposure Variables.

### 2.2. Information sources

The search was carried out using the electronic databases Web of Science (WoS), Scopus, Cochrane Central Register of Controlled Trials (CENTRAL) and PsycInfo. The search was closed on 23rd November 2021. An update of the search was conducted on the 4th of October 2023.There were no limits regarding the publication year. The only limitation imposed on the search was the language, including only documents in English or Spanish.

### 2.3. Search strategy

In consideration of the format of each database, the following keywords in English were used in combination with the Boolean logic operators: (“eating disorders” OR “anorexia” OR “bulimia” OR “binge eating” OR “binge eating disorder” OR “unspecified eating disorder”) AND (impuls* OR “emotional dysregulation” OR “social media exposure” OR “social media misuse” OR “social media use” OR “perfecctio*” OR “self-demand” OR “self-esteem” OR “social support” OR “maturity fear*” OR “expressed emotion”) AND (“high school” OR adoles* OR teen*).

### 2.4. Data extraction and coding

All identified documents were imported into Rayyan, a research tool designed to work with systematic reviews, to eliminate duplicates. The screening process was performed by three independent reviewers, and disagreements were solved by discussion. If consensus was not achieved, the reviewer with the most experience in the area made the final decision. Data extraction was conducted by the same independent reviewers. The extracted information includes the following: (a) study identification: authorship, year, and country; (b) characteristics of the participants: sample size, gender and age; (c) characteristics of the exposure variables mentioned above, types and measuring instrument; (d) characteristics of the outcome variable: measuring instrument e) characteristics of statistical analysis: type and results.

### 2.5. Quality and risk of bias assessment

The methodological quality of the selected articles was assessed by four independent reviewers using the tool recommended by Cochrane, Quality in Prognosis Studies (QUIPS; Grooten et al., 2019). To specifically analyze the risk of bias, six main domains were evaluated: (i) study participation; (ii) study attrition; (iii) prognostic factor measurement; (iv) outcome measurement; (v) study confounding; and (vi) statistical analysis and reporting.

Several descriptors are presented in each domain, which were ranked on a three-point scale (high, medium, or low level of risk), according to the tool specification, assess the risk of potential bias in the results (Grooten et al., 2019).

## 3. Results

**Figure 1:**
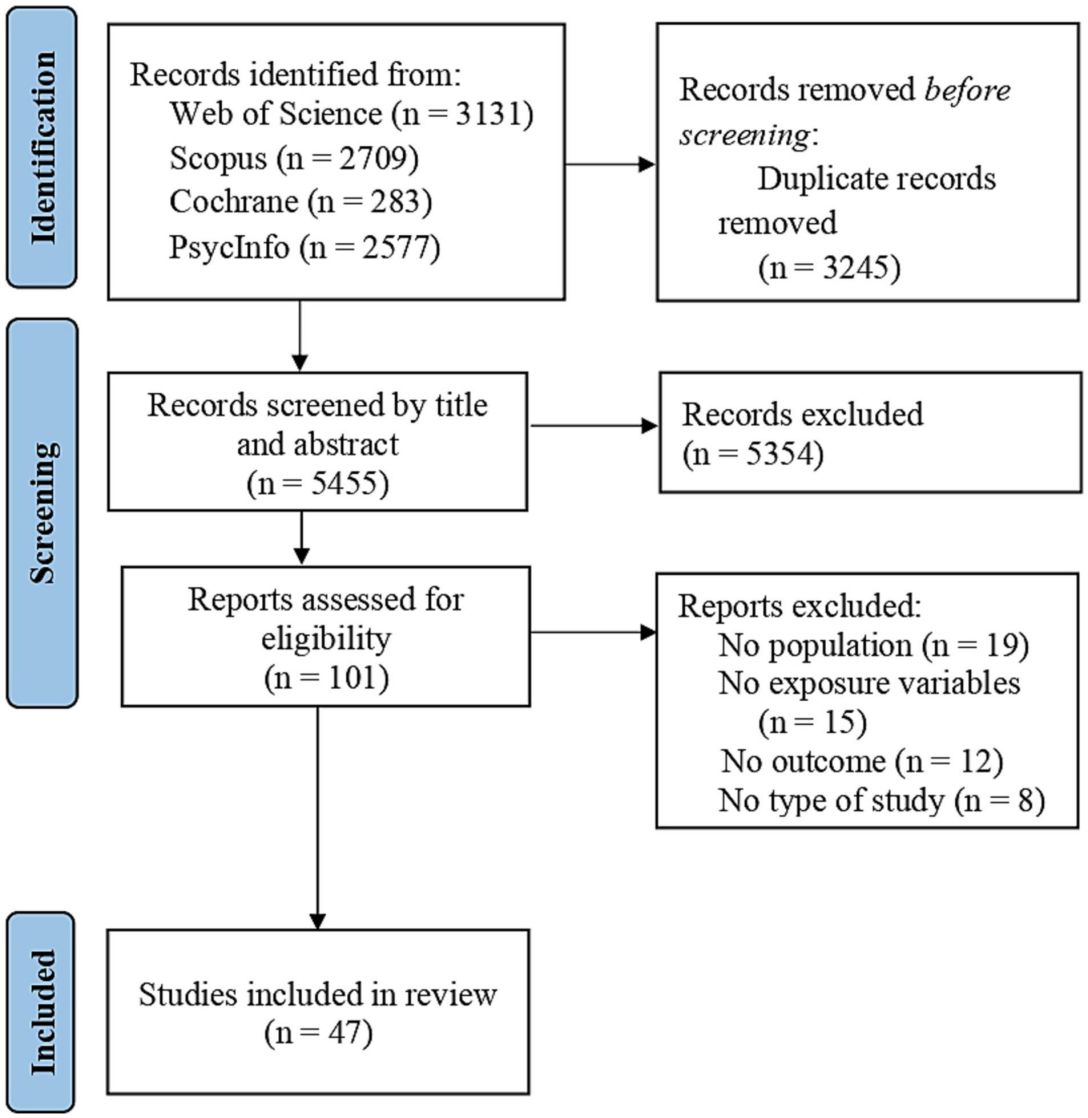
Flow diagram of study selection.

The initial number of identified articles was 8,700, including the initial search and the update. After removing duplicates, 5,455 studies were screened by three independent reviewers and 101 were selected for full-text screening. Finally, 47 studies met the pre-specified criteria and were included in the systematic review (Figure 1).

### 3.1. Descriptive data

A total of 47 studies were finally included in this systematic review after meeting the pre-specified inclusion criteria. These articles were conducted from 1996 to 2023 to cover the widest possible range and provide in-depth analysis of eating disorder predictors. The total sample comprised 41,115 teenagers, mean age = 14.9 years and ranged from 11 to 19 years. Most of the sample were women, 17 studies presented 100% women, 18 studies ≥50% women, 11 studies <50% women and only 1 study 100% men.

The studies were carried out in the United States (n = 6), Australia (n = 4), Spain (n = 5), Italy (n = 3); China, Iran, Belgium, Portugal, Brazil, Mexico, United Kingdom, Greece, Cyprus (each country, *n* = 2); Chile, Germany, Israel, Canada, Thailand, New Zealand, Malaysia, Netherlands, India, Bosnia Herzegovina and Turkey (each country, n = 1). Almost 70% of the studies (n = 33) were carried out in countries considered Western Societies. However, the risk factors identified in the included studies did not present relevant differences in terms of country or culture. All studies had a cross-sectional design except for eight longitudinal studies (Shomaker and Furman, 2009; Bachar et al., 2010; Boone et al., 2014; Dakanalis et al., 2014; Wade et al., 2015; Pace et al., 2018; Evans et al., 2019; Beckers et al., 2023). Most of the studies used regression and structured equation modeling analysis. The number of participants selected in longitudinal studies were indicated by the authors to carry out the statistical analysis indicated in Table 2.

**Table 2:** Summary table of the included studies.

Author, year, and country	*N*	Age (M, SD/Range)	Female (%)	Exposure variables	Outcome variables	Statistical analysis	Results	Conclusions
Altamirano et al. (2011) Mexico	1982	16.3 (1.0) 15–19	100	Self-esteem Scale Figure Rating Scale	BQREB	Logistic multinomial Regression	Females High Risk Eating Disorder: Higher Body dissatisfaction (OR = 2.0, 95%CI [1.3–3.0], *p* < 0.001) and Self-esteem (OR = 1.2, 95%CI [1.2–1.3], *p* < 0.001) than females with no risk of eating disorder. Females Moderate Risk Eating Disorder: Higher Body dissatisfaction (OR = 1.8, 95%CI [1.5–2.2], *p* < 0.001) and Self-esteem (OR = 1.1, 95%CI [1.1–1.2], *p* < 0.001) than females with no risk of eating disorder.	Body dissatisfaction and low Self-esteem were predictive factors of the onset of eating disorders for female teenagers.
Argydes et al. (2020) Cyprus	2,605	15.2(1.2) 13–16	59.2	MBSRQ-AS RSES SATAQ-3 BAS-2^1^	EAT-26	Stepwisemultiple regression analyses	Female Models Overweight Preoccupation → EAT-26 (*R*^2^ = 0.33, *β* = 0.32, *p* < 0.001); Body Appreciation → EAT-26 (*R*^2^ = 0.46, *β* = −0.35, *p* < 0.001); Body Dysphoria → EAT-26 (*R*^2^ = 0.50, *β* = 0.17, *p* < 0.001); Media Influence → EAT-26 (*R*^2^ = 0.51, *β* = 0.12, *p* < 0.001) Male Models Overweight Preoccupation → EAT-26 (*R*^2^ = 0.40, *β* = 0.10, *p* < 0.001);Body Appreciation → EAT-26 (*R*^2^ = 0.35, *β* = −0.51, *p* < 0.001); Body Dysphoria → EAT-26 (*R*^2^ = 0.39, *β* = 0.11, *p* < 0.001); Body Satisfaction → EAT −26 (*R*^2^ = 0.41, *β* = −0.06, *p* < 0.05); Media Influence → EAT-26 (*R*^2^ = 0.41, *β* = 0.09, *p* < 0.01)	The findings emphasized the role of weight/ appearance-related anxiety and situational body image dysphoria as the most significant risk factors in the development of eating disorders in both male and female adolescents.
Bachar et al. (2010). Israel	114	16.1 (0.52) 15–16	100	Selflessness Scale MPS	EAT-26	Hierarchical regression analysis	Selflessness at 7th grade → EAT-26 at 10th grade (*R*^2^ = 0.05, *p* < 0.001) EAT-26 at 7th grade → EAT-26 at 10th grade (*R*^2^ = 0.36, *p* < 0.001)	Students who substantially increased their scores for EAT-26 from 7th grade to 10th grade, also presented higher scores for Selflessness at 10th grade. Perfectionism did not find to predict any eating behaviors.
Bacopoulou et al. (2017) Greece	90	14.0 (1.8)	73.3	YSR	EAT-26	Multivariate Linear regression analysis	YSR Anxiety was a predictor of negative eating behaviors (*b* = 0.30, *p* = 0.04). Gender moderates the relationship between YSR anxiety and eating behaviors (*b* = 0.59, *p* = 0.01). This effect held true for bulimia subscale (*b* = 0.20, *p* = 0.03) but not for the other subscales diet and oral control.	Elevated anxiety levels were a risk factor for disordered behaviors, especially bulimic symptomatology in girls.
Baylan et al. (2009) Turkey	1,201	15.2 (0.42) 14–16	100	BDI RSES Obsessive Compulsive Subscale (SCL-90-R) FAD BPS	-EAT -BITE	Multiple regression analysis	BITE Model, the variables presented significant associations were: Obsessive Compulsive Subscale (*R*^2^ = 0.12, *F* = 153.05, *β =* 0.341, *p* < 0.000); Depression (*R*^2^ = 0.13, *F* = 23.98, *β =* 0.162, *p* < 0.000); Affective Involvement (FAD) (*R*^2^ = 0.16, *F* = 12.87, *β =* 0.104, *p* < 0.000) EAT Model, the variables presented significant associations were: Affective Involvement (FAD) (*R*^2^ = 0.19, *F* = 134.8, *β =* 0.135, *p* < 0.000); Obsessive Compulsive Subscale (*R*^2^ = 0.22, *F* = 83.1, *β =* 0.129, *p* < 0.000); Depression (*R*^2^ = 0.16, *F* = 221.8, *β* = 0.277, *p* < 0.000); Problem Solving (FAD) (*R*^2^ = 0.23, *F* = 70.0, *β = −* 0.134, *p* < 0.000); Roles (FAD) (*R*^2^ = 0.24, *F* = 59.3, *β* = *0*.078, *p* < 0.000)	The predictors of anorectic and bulimic symptoms were similar (obsessive compulsive symptoms, depression, and family involvement). However, obsessive compulsive symptoms were stronger for bulimia, meanwhile depression was more present for anorexia.
Beckers et al. (2023) Netherlands	2051	13.8 (0.72) 11.4–16.9	48.5	OBVQ RSES Negative Affectivity Subscale (Type D Scale 14)	-DEBQ -EDI (overeating items)	SEM	In any model Interpersonal peer problems (Time 1) were significantly associated with Self-Esteem or Negative Affect (Time 2). Full Model 1 Overeating. (TLI = 0.959, CFI = 0.988, RMSEA = 0.035): -Self-esteem (Time 2) ➔ Overeating (Time 3) (*β* = −0.141, *p* < 0.001) Full Model 2 Emotional eating. (TLI = 0.964, CFI = 0.990, RMSEA = 0.034): -Self-esteem (Time 2) ➔ Emotional Eating (Time 3) (*β* = −0.158, *p* < 0.001) Full Model 3 Restrained eating: TLI = 0.955, CFI = 0.987, RMSEA = 0.040). -Self-esteem (Time 2) ➔ Restrained eating (Time 3) (*β* = −0.345, *p* < 0.001) -Interpersonal Peer Problems (Time 1) ➔ Restrained eating (Time 3) (*β* = −0.214, *p* < 0.001)	More research is needed to support the mediator role of self-esteem and negative affectivity between interpersonal peer problems and disordered eating. However, self-esteem showed a significant and stronger association with the three types of subsequent disordered eating behaviors than negative affectivity.
Boone et al. (2014) Belgium	455	13.3 (0.85) 12–15	100	F-MPS Body Dissatisfaction subscale (EDI-2)	EDI-2 EDEQ	Regression analysis	Full Model Drive for Thinness. (*R*^2^ = 0.01, *p* < 0.01). Predictors: Body Dissatisfaction (*β* = 0.11, *p* < 0.05); Personal Standards Perfectionism × Body Dissatisfaction (*β* = 0.09, *p* < 0.01) Full Model Bulimic Symptoms (*R*^2^ = 0.01, *p* < 0.05). Predictors: Evaluative Concerns Perfectionism (*β* = 0.10, *p* < 0.05) Full Model Over Evaluation Weight and Shape (*R*^2^ = 0.09, *p* < 0.001). Predictors: Evaluative Concerns Perfectionism (*β* = 0.15, *p* < 0.001); Body Dissatisfaction (*β* = 0.33, *p* < 0.001) Full Model Over Evaluation Weight and Shape (*R*^2^ = 0.01, *p* < 0.01). Predictors: Evaluative Concerns × Body Dissatisfaction (*β* = 0.11, *p* < 0.001)	Body dissatisfaction moderated some of the effects of perfectionism on changes in eating disorder symptoms. Concretely, personal standards of Perfectionism for Drive for Thinness and, Evaluative Concerns for Over Evaluation Weight and Shape.
Cella et al. (2021) Italy	1,046	14.4 (1.5) 11–19	45.1	RSES BIS	BES	Path Model analysis	Females model 27% variance in binge eating. Direct effect self-esteem – binge eating (*β =* −0.395, *p* < 0.001) Indirect effect self-esteem -binge eating (*β =* −0.153, *p* = 0.01). Mediators: body image feelings (*β =* −0.202, 95%CI [−0.276, −0.130] *p* < 0.001) and body protection (*β =* −0.064, 95%CI [−0.096, −0.037] *p* < 0.001) Males model 26% variance in binge eating. Direct effect self-esteem – binge eating (*β =* −0.272, *p* < 0.001) Indirect effect self-esteem -binge eating (*β =* −0.034, *p* = 0.5). Mediators: body image feelings (*β =* −0.158, 95%CI [−0.225, −0.096] *p* < 0.001) and body protection (*β =* −0.044, 95%CI [−0.072, −0.025] *p* = 0.001)	These results show that reduced body protection and negative body feelings mediate the relationship between self-esteem and binge eating both female and male
Ćorić et al. (2023) Bosnia-Herzegovina	724	16.7 (1.1) 14–19	62.7	BAS^2^ RSES KOBI	EAT-26	Multiple regression analysis	Female Model EAT-26. (*R*^2^ = 0.236, *p* < 0.01). Predictors: Body Appreciation (*β = −*0*-*379, *p* = 0.001) Female Model EAT-26. (*R*^2^ = 0.125, *p* < 0.01). Predictors: Body Appreciation (*β* = −0.199, *p* = 0.010), Self-esteem (*β* = −0.211, *p* < 0.022)	The most significant risk factors for developing disordered eating in adolescents are body appreciation and self-esteem
Costarelli et al. (2011) Greece	202	16.7 (0.60) 15–18	46	SPPA IECA STAI Overweight Preoccupation Subscale (MBSRQ)	EAT-26	Binary Regression analysis	Anxiety levels → EAT-26 (*β =* 0.30, *p* = 0.019) Perceived Physical Appearance → EAT-26 (*β = −*1.269, *p* = 0.01)	Higher scores on anxiety levels and lower punctuations on perceived physical appearance were the two variables found as predictors of developing eating disorders.
Cruz-Sáez et al. (2018) Spain	806	16.8 (0.83) 16–19	61.8	Body dissatisfaction subscale (EDI-2) Negative self-beliefs subscale (EDBQ) Anxiety and Depression subscales (GHQ)	-Drive for thinness subscale (EDI-2) -Bulimia subscale (EDI-2)	Mediation model	Female Model. Significant effect of body dissatisfaction on Disordered eating (*B* = 0.83, *p* < 0.001). Also, significant effect of body dissatisfaction through negative affect on disordered eating (*B* = 0.042, Boot SE = 0.013, 95% CI = 0.021–0.071). Finally, sequential indirect effect of body dissatisfaction through negative self-esteem and negative affect (*B* = 0.057, Boot SE = 0.012, 95% CI = 0.036–0.085). Male Model. Significant effect of body dissatisfaction on Disordered eating (*B* = 0.68, *p* < 0.001). Also, significant effect of body dissatisfaction through negative affect on disordered eating (*B* = 0.020, Boot SE = 0.013, 95% CI = 0.002–0.056). Finally, sequential indirect effect of body dissatisfaction through negative self-esteem and negative affect (*B* = 0.036, Boot SE = 0.015, 95% CI = 0.012–0.071).	Body dissatisfaction presented both indirect and direct effects on disordered eating. However, the role of negative affect and self-esteem as mediators of this relationship was significant to both girls and boys.
Dakanalis et al. (2014) Germany	627	14.5 (0.28) 14–15	52.1	General and Athletic Internalization Subscales (SATAQ-3) internalization Body Surveillance subscale (OBCS) Body shame subscale (OBCS) SAAS K-SADS-P	EDE OBE	SEM	Full Model. (*X*^2^_*(df* = 317)_ = 601.65, *p* < 0.001; CFI = 0.97; SRMR = 0.06) Female/Male Models. Indirect Effects EDE: -Media-ideal Internalization → Self-Objectification → Body Shame (*β =* 0.11/0.10, *p* < 0.05) -Self-Objectification → Body Shame → EDE (*β =* 0.05/0.04, *p* < 0.05) -Media-ideal Internalization → Self-Objectification → Appearance Anxiety (*β =* 0.06/0.05, *p* < 0.05) -Self-Objectification → Appearance Anxiety → EDE (*β =* 0.03/0.03, *p* < 0.05) Female/Male Models. Indirect Effects OBE: -Media-ideal Internalization → Self-Objectification → Body Shame (*β =* 0.11/0.10, *p* < 0.05) -Self-Objectification → Body Shame → OBE (*β =* 0.08/0.07, *p* < 0.05) -Media-ideal Internalization → Self-Objectification → Appearance Anxiety (*β =* 0.06/0.05, *p* < 0.05) -Self-Objectification → Appearance Anxiety → OBE (*β =* 0.04/0.03, *p* < 0.05)	Regardless of gender, self-objectification (via body surveillance) may serve as a mechanism which translates the media-ideal internalization into negative body-feelings. Body shame and appearance anxiety may constitute the mechanisms through which thinking and scrutinizing of one’s own body from an external observer’s perspective contributes to dietary restraint and binge eating.
Evans et al. (2019) United States	238	13.0 (0.89) 11–15	46	PANAS-C EI-7	COEDS	Latent growth curve	Following the full model (*X*^2^_*(df* = 41)_ = 69.6, *p* = 0.004; CFI = 0.96; TLI = 0.96; RMSEA = 0.05). Negative affect (*b =* 0.15, *p* < 0.05), impulsivity (*b =* 0.21, *p* < 0.01) and gender (*b =* −0.40, *p* < 0.01) were significantly associated with ED-Attitudes at baseline. Impulsivity × negative affect was significantly associated with the growth curve of ED-Attitudes (*b =* −0.60, *p* < 0.05) for high negative affect reactivity group.	These results suggest that higher levels of impulsivity and affect reactivity were identified as risk factors for the development of ED-Attitudes
Gan et al. (2018) Malaysia	356	14.3(1.0) 13–16	57.3	RSES FES CES-D Contour Drawing Rating Scale	BES	Multiple linear regression analysis	Prediction Model (*R*^2^ = 0.17, *F* = 15.056, *p* < 0.001). Predictors: Depressive symptoms (*β =* 0.19, *p* < 0.001); Family Cohesion (*β =* −0.21, *p* < 0.001); Perceptions of body size (*β =* 0.16, *p* = 0.002) and self-esteem (*β =* −0.15, *p* = 0.003) contributed significantly to binge eating behaviors	High levels of depressive symptoms, high levels of body dissatisfaction, low levels of self-esteem and low levels of family cohesion have been identified as predictive factors of binge eating symptomatology
García-Grau et al. (2002) Spain	216	15.9 (1.5) 14–18	100	ACS	EDI-2	Hierarchical regression model	Intropunitive avoidance (*R^2^* = 0.29; *β =* 0.54, *p* < 0.001), Problem-focused action (*R^2^* = 0.30; *β = −*0.15, *p* < 0.01) and Avoidance of social support (*R^2^* = 0.32; *β =* 0.14, *p* < 0.05) showed a significant association with the risk of suffer an eating disorder	Passive coping strategies like intropunitive avoidance and avoidance of social support presented positive and significant associations with EDI-2 total score. However, active coping strategies like problem-focused action presented negative associations with EDI-2 total score.
Garrusi et al. (2016) Iran	433	15.9 (0.80) 14–18	0	BSI Socio-cultural pressure instrument RSES	EDDS	Logistic regression model	Body Dissatisfaction → EDDS (OR = 1.23, 95%CI: 1.01 to 1.50, *p* = 0.04) One unit increase in body dissatisfaction score was associated with 23% increase in risk of eating disorders.	Body dissatisfaction was the only psychological variable significantly associated with the risk of developing an eating disorder.
Gomes et al. (2015) Portugal	192	15.6 (1.4) 13–18	53.1	Dieting status measure PAQ-A Goal orientation in exercise measure SPAS-R RSES	EDE-Q	Regression analysis with blocked entry procedures	Exercise Frequency → EDE-Q (*R*^2^ = 0.03, *F*_(1, 173)_ = 5.20, *β =* 0.17, *p* < 0.05); Desire Ideal Weight → EDE-Q (*R*^2^ = 0.45, *F*_(4, 170)_ = 34.02, *β = −*0.56, *p* < 0.001); Psychological dimensions → EDE-Q (*R*^2^ = 0.76, *F*_(9, 165)_ = 56.47, *p* < 0.001); Social Physique Anxiety (*β =* 0.48, *p* < 0.001); Self-esteem (*β = −*0.19, *p* < 0.01)	Regular exercise seems to be associated with psychological well-being and with a lower propensity for eating disordered behaviors in adolescents.
Halliwell and Harvey (2006) United Kingdom	507	13.2 (1.6) 11–16	49.3	MBSRQ SATAQ PSPS	ChEAT	SEM	Female Model. (*X*^2^_*(df* = 2)_ = 6.06, *p* = 0.05; CFI = 0.93; SRMR = 0.03). Indirect Effect. Weight pressure → Eating Behaviors. Mediators: Social Comparisons (*b* = 0.19, *p* < 0.05) → Internalization (*b* = − 0.14, *p* < 0.05) → Body Dissatisfaction (*b* = 0.13, *p* < 0.05). Male Model. (*X*^2^_*(df* = 3)_ = 6.79, non-significant; CFI = 0.98; SRMR = 0.04). Indirect Effect. Weight pressure → Eating Behaviors. Mediators: Social Comparisons (*b* = 0.12, *p* < 0.05) → Internalization (*b* = − 0.06, *p* < 0.05) → Body Dissatisfaction (*b* = 0.06, *p* < 0.05).	Pressure to be thin was associated with eating behaviors. This relationship was moderated by social comparisons, internalization of thin ideals and body dissatisfaction. High scores on social comparisons were the most strongly related mediator. The pressure to be thin and develop an eating disorder is higher in adolescents with higher scores in social comparisons.
Iannaccone et al. (2016) Italy	222	15.5 (1.5) 13–19	38.7	PBI RSES ESS MPS	EDRC	Hierarchical regression model	Following the full model for participants with obesity (*R^2^* = 0.39; *F*_(14, 96)_ = 5.97; *p* < 0.001) Experienced body shame was the only variable significantly associated with EDRC (*β =* 0.43, *p* < 0.01). Body shame mediates the association between self-esteem and EDRC. Self-esteem and body shame (*β =* −0.54, *p* < 0.001), body shame and EDRC (*β =* 0.53, *p* < 0.001) Following the full model for participants with normal weight (*R^2^* = 0.48; *F*_(14, 96)_ = 8.16; *p* < 0.001). The variables significantly associated with Eating Disorder Risk were gender (*β =* 0.22, *p* < 0.05), BMI (*β =* 0.31, *p* < 0.001), maternal care (*β =* −0.24, *p* < 0.01) and experienced body shame (*β =* 0.47, *p* < 0.001). Body shame mediates the association between self-esteem and EDRC. Self-esteem and body shame (*β =* −0.54, *p* < 0.001), body shame and EDRC (*β =* 0.57, *p* < 0.001)	Body shame presented the strongest relationship with eating disorder risk for both groups, acting as a mediator between low self-esteem and eating disorder risk.
Jones et al. (2020) Australia	270	14.9 (0.83) 13–18	95.2	Interpersonal Problems Subscale (EDI-3) Affective Problems Subscale (EDI-3) Perfectionism Subscale (EDI-3) RSES	Child- EDE	Pathway analysis	Full Model. (*X*^2^ = 1.02, *p* < 0.001; CFI = 1; SRMR = 0.009) Indirect Effects through Self-esteem between: -Perfectionism → Eating Concerns (*b =* 0.16, *p* < 0.001); Perfectionism → Weight Concerns (*b =* 0.29, *p* < 0.001); -Perfectionism → Shape Concerns (*b =* 0.29, *p* < 0.001); -Perfectionism → Dietary Restraint (*b =* 0.23, *p* < 0.001) Indirect Effects through mood intolerance between: -Perfectionism → Eating Concerns (*b =* 0.27, *p* < 0.001); Perfectionism → Weight Concerns (*b* = 0.17, *p* = 0.003); Perfectionism → Shape Concerns (*b =* 0.08, *p* = 0.047); Perfectionism → Dietary Restraint (*b* = 0.14, *p* = 0.021)	Low self-esteem and mood intolerance were directly associated with eating disorder symptoms. Perfectionism was indirectly associated with eating disorder symptoms through self-esteem, and mood intolerance. There were no significant associations for interpersonal difficulties.
Kaewpradub et al. (2017) Thailand	620	15.7 (1.9)	60.3	RSES Social media and internet use/behaviors (*ad hoc*) BESAA	EAT-26	Multiple regression analysis	Internet use in relation to eating problems (OR = 1.13, 95%CI = 1.08–1.17, *p* < 0.001) Internet use in relation to binge eating (OR = 1.05, 95%CI = 1.01–1.08, *p* < 0.01) Internet use in relation to purging behavior (OR = 1.10, 95%CI = 1.06–1.14, *p* < 0.001) Internet use in relation to taking laxatives (OR = 1.06, 95%CI = 1.04–1.09, *p* < 0.001) Social network use to eating problems (OR = 1.07, 95%CI = 1.04–1.09, *p* < 0.001) Social network use to binge eating (OR = 1.03, 95%CI = 1.01–1.05, *p* < 0.05) Social network use to purging behavior (OR = 1.06, 95%CI = 1.04–1.09, *p* < 0.05) Social network use taking laxatives (OR = 1.05, 95%CI = 1.03–1.08, *p* < 0.05)	Time spent on Internet and using social media were associated with different types of eating problematic behaviors
Kerremans et al. (2010) Belgium	339	16.8 (1.3) 14.3–19.5	64.9	BIS/BAS^1^ EBP SPPA	EDI-2	Regression analysis	Female model. Personal Variables: Bulimia (*R^2^* = 0.31). Predictors: Interoceptive Awareness (*β =* 0.55, *p* < 0.001). Male model. Personal Variables: Bulimia (*R^2^* = 0.21). Predictors: Interoceptive Awareness (*β =* 0.43, *p* < 0.001). Female model. Temperament Variables: Bulimia (*R^2^* = 0.16). Predictors: Behavioral Inhibition System (*β =* 0.26, *p* < 0.01), Effortful Control (*β = −*0.35, *p* < 0.01). This model did not present significant associations for men. Female model. Depressive symptoms and Antisocial behavior: Bulimia (*R^2^* = 0.21). Predictors: Depressive symptomatology (*β = 0*.40, *p* < 0.01). Male model. Depressive symptoms and Antisocial behavior: Bulimia (*R^2^* = 0.35). Depressive Symptomatology (*β =* 0.26, *p* < 0.01), Covert Delinquency (*β =* 0.46, *p* < 0.01). Female model Self-Competence: Bulimia (*R^2^* = 0.23). Global Self-Esteem (*β =* 0.28, *p* < 0.05). This model did not present significant associations for men.	These series of regression analysis showed that the variables mainly related with the onset of bulimia were interoceptive awareness, behavioral inhibition system, effortful control, depressive symptoms, and global self-esteem for female. For males, these variables were interoceptive awareness, depressive symptoms, and covert delinquency.
Koushiou et al. (2020) Cyprus	418	13.7 (1.0) 13–15	55.9	RSES AFQ-Y8	EAT-26	Mediation analysis	The amount of variance in eating disorder explained by psychological inflexibility and self-esteem was 22.25% (*F*_(2, 415)_ = 59.37, *p* < 0.001) Self-esteem was related with Psychological Inflexibility (*b* = −0.60, *p* < 0.001). Psychological inflexibility was related with Eating Pathology (*b* = 0.54, *p* < 0.001)	The relationship between self-esteem and eating pathology was partiality mediated by psychological inflexibility.
Lee et al. (2018) United States	158	15.1 (2.2) 14–18	57.0	FACES-IV Momentary Moods	Binge Eating Subscale (EDDS)	Mediation model	Tiredness → Binge Eating. Mediators: Family Cohesion (*β* = − 0.16, *p* < 0.05); Family satisfaction (*β* = −0.12, *p* < 0.05); Family Balance (*β* = −0.71, *p* < 0.01) Stress → Binge Eating. Mediators: Family Balance (*β* = −0.91, *p* < 0.05) Left-out → Binge Eating. Mediators: Family Cohesion (*β* = 0.52, *p* < 0.05) Happiness → Binge Eating. Mediators: Family Cohesion (*β* = 0.019, *p* < 0.05) Embarrassed → Binge Eating. Mediators: Family Cohesion (*β* = 0.047, *p* < 0.05) Boredom → Binge Eating. Mediators Family Cohesion (*β* = −0.014, *p* < 0.05)	Momentary moods like stress, frustration, boredom, tiredness, or negative affect were significantly associated with binge eating measures. Family variables, especially, family cohesion were mediators of these associations.
Lee-Win et al. (2016) USA	10,028	15.3 (1.5) 13–18	50.8	ZKPQ WOCS	CIDI	Regression analysis	Escape-avoidance coping in relation to lifetime binge eating disorder (OR = 1.13, 95%CI = 1.10–1.18, *p* < 0.001)	Lifetime prevalence of binge eating was 1.13 times higher with escape-avoidance coping strategy. The other coping styles or personality traits did not present significant associations with binge eating.
Leung et al. (1995) Canada	918	14.6 (1.4) 12–17	100	FACES-III RSES Body Dissatisfaction Subscale (EDI) FNE	EAT-26	SEM	Following the full model (*X*^2^_*(df* = 40)_ = 353.47, *p* < 0.001; CFI = 0.92; NFI = 0.92). Family preoccupation with weight and appearance had direct effects (*b* = 0.32, *p* < 0.001) on negative eating behaviors. Also, indirect effects through body dissatisfaction (*b* = 0.40, *p* < 0.001) with negative eating behaviors (*b* = 0.17, *p* < 0.001). Family preoccupation with weight and appearance effects through body dissatisfaction were also mediated by self-esteem deficit (*b* = 0.11, *p* < 0.001) on negative eating behaviors. Family functioning presented an indirect effect, mediated by self-esteem (*b* = −0.39, *p* < 0.001), on negative eating behaviors (*b* = −0.09, *p* < 0.001)	Family preoccupation with weight and appearance and family functioning were related to negative eating behaviors. These associations were mediated by body dissatisfaction and low self-esteem.
Li and Li (2021) China	256	15.4 (1.4) 13–18	40.6	BESAA WLEIS	EAT-26	Hierarchical multiple regressions	Body-esteem → Eating Disorder Risk (*R*^2^ = 0.45, *p* < 0.001). Predictors: Body-esteem (*β* = −. 35, *p* < 0.001), Emotional Intelligence was identified as moderator (*β* = −0.017, *p* < 0.01)	Body-esteem leads to emotional intelligence. The relationship between body-esteem and eating disorder risk was stronger with higher scores of emotional intelligences.
Macedo-Uchôa et al. (2019) Chile	1,011	15.7 (1.1) 14–18	52.1	SATAQ-3 BSQ	EAT-26	Hierarchical multiple linear regression	Female Model. (*R*^2^ = 0.44, *F*_(1, 525)_ = 404.71, *p* = 0.000). Predictors: Body Dissatisfaction (*β* = 0.660, t = 20.12, *p* = 0.000) Male Model. (*R*^2^ = 0.36, *F*_(2, 418)_ = 135.20, *p* = 0.000). Predictors: Body Dissatisfaction (*β* = 0.539, *t* = 13.20, *p* = 0.000), Mass Media Influence (*β* = 0.119, *t* = 2.94, *p* = 0.003)	Body dissatisfaction was found as a predictor of eating disorders for both genders. Mass media influence was found as a predictor of ED only for boys. For girls, SATAQ-3 was not found as a predictor and was excluded from the model.
McCabe and Vincent (2003) Australia	603	13.8 (1.1) 11–17	50,7	RSES DASS Ineffectiveness and Perfectionism subscales (EDI) PDS	BULIT-R	Multiple regression analysis	Females. -Model for Extreme Weight Loss (*R*^2^ = 0.12, *F*_(8, 299)_ = 4.47, *p* < 0.001). Anxiety (*β* = 0.24, *p* = 0.03). -Model for Binge Eating (*R*^2^ = 0.37, *F*_(8, 299)_ = 18.6, *p* < 0.001). Self-esteem (*β* = 0.21, *p* = 0.02) and depression as significant predictors (*β* = 0.23, *p* = 0.02). -Model for Bulimic Symptoms (*R*^2^ = 0.40, *F*_(8, 299)_ = 25.98, *p* < 0.001). Self-esteem (*β* = 0.25, *p* = 0.02) and depression as significant predictors (*β* = 0.24, *p* = 0.02). Males. -Model for Extreme Weight Loss (*R*^2^ = 0.09, *F*_(8, 292)_ = 3.03, *p* < 0.001). Ineffectiveness (*β* = 0.16, *p* = 0.02) and Anxiety (*β* = 0.17, *p* = 0.02) as significant predictors. -Model for Binge Eating (*R*^2^ = 0.31, *F*_(8, 292)_ = 14.5, *p* < 0.001). Self-esteem (*β* = 0.17, *p* = 0.02), Anxiety (*β* = 0.27, *p* = 0.04) and Perfectionism as significant predictors (*β* = 0.13, *p* = 0.02). -Model for Bulimic Symptoms (*R*^2^ = 0.40, *F*_(8, 292)_ = 18.36, *p* < 0.001). Anxiety (*β* = 0.16, *p* = 0.02) and Ineffectiveness as significant predictors (*β* = 0.28, *p* = 0.02).	There were differences between the predictors of negative eating behaviors between girls and boys. The predictors for girls were anxiety, self-esteem, and depression. For boys, the associated variables were ineffectiveness, anxiety, self-esteem, and perfectionism.
Mora et al. (2022) Spain	579	13.7 12–16	42.7	RSES	EAT-26	Logistic regression analysis	Self-esteem ➔ Disordered Eating Behaviors (OR = 0.91; 95% CI 0.88–0.94: *p* < 0.001).	Subjects with higher self-esteem have a lower risk of developing EDs. Per each increase of one point in the self-esteem dimension, the risk of belonging to the risk group for eating disorders was reduced by 9.0%
Pace et al. (2018) Italia	482	17.9 (0.57) 17–18	49.2	DAPCS SSQ	EAT-26	Regression analysis	Final model (*R*^2^ = 0.24, *F*_(1, 477)_ = 20.93, *p* < 0.001). Predictors: Paternal achievement oriented psychological control (*β* = 0.38, *p* < 0.001), perceived peer support (*β* = −0.21, *p* < 0.001) and the interaction of these variables (*β* = −0.34, *p* < 0.000)	Results showed that peer perceived support was a moderator in the relationship between father’s psychological control and negative eating attitudes and behaviors.
Pamies-Aubalat et al. (2022) Spain	1,630	14 (1.34) 12–18	55	RSES CAPS Body Dissatisfaction subscale (EDI-2) DCS Pressure from significant others to lose weight	EAT-40	Logistic regression analysis	Female Model (*R*^2^ = 74.2%,) Predictors: Diet × Body Dissatisfaction × Affective Social Comparison (OR = 3.772, 95%CI = 2.08–6.82, *p* < 0.001) Male Model (*R*^2^ = 48.4%,) Predictors: Body Dissatisfaction × Pressure to lose weight (OR = 3.282, 95%CI = 1.94–5.54, *p* < 0.001)	Girls who experienced dieting, body dissatisfaction and social comparison together are 3.8 times more likely to have disordered eating attitudes. In the model for the boys, the odds ratio indicated that boys who experienced body dissatisfaction and the pressure to lose weight together are 3.3 times more likely to have disordered eating attitudes
Piko et al. (2023) India	112	16.01 (1.08) 14–18	47.3	SAS-SV K-GSADS-A MSPSS	EAT-26	Binary logistic regression analysis	Full Model (χ2 = 34.72, df = 10, *R*^2^ = 0.60) Predictors for EAT-26: Smartphone Addiction (OR = 1.07, 95% CI = 1.01–1.14, *p* < 0.01); Social Anxiety (OR = 1.05, 95% CI = 1.01–1.10, *p* < 0.01); Social Avoidance (OR = 1.07, 95% CI = 1.02–1.13, *p* < 0.01); Social Support (OR = 0.95, 95% CI = 0.92–0.98, *p* < 0.01)	Adolescents who presented smartphone addiction were more likely to present social anxiety and social avoidant. These participants were more likely to present disordered eating behavior. Social support was identified as a protective factor.
Rodgers et al. (2014) Australia	488	12.4 (0.53) 12–13	100	CDI-SF RSES PSPS PWT Internalization of thin ideal subscale (SATAQ) Body dissatisfaction subscale (EDI) Shape and Weight Concerns subscale (EDEQ) Restrained eating behavior subscale (DEBQ) Appearance Comparison	Bulimia Scale of EDI	SEM	Following the full model (*X*^2^_*(df* = 37)_ = 148.4, *p* < 0.001; CFI = 0.962; RMSEA = 0.080). Negative affected (*b* = 0.36, *p* < 0.01) and Sociocultural influence (*b* = 0.71, *p* < 0.01) were related with internalization and comparison. The last one was related with body image concerns (*b* = 0.79, *p* < 0.01) and finally, this variable presented an association with bulimia symptoms (*b* = 0.14, *p* < 0.05). At the same time, negative affect presented a direct association with bulimic symptoms (*b* = 0.37, *p* < 0.01).	The relationship between negative affect and social cultural influence with bulimic symptoms was mediated by internalization and comparison and body image concerns. However, negative affect also presented a direct effect on bulimic symptoms.
Rosewall et al. (2018) New Zealand	231	15.5 (1.1) 14–18	100	CAPS RSES PANAS POTS Perceive pressure to lose weight (SIBIBCQ)	EAT-26	Regression analysis	Moderation effects between body dissatisfaction and eating pathology for the following variables: Self-oriented perfectionism (*R*^2^ = 0.08, *b* = 0.41, *p* < 0.001); Socially prescribed perfectionism (*R*^2^ = 0.06, *b* = 0.37, *p* < 0.001); Self-esteem (*R*^2^ = 0.02, *b* = 0.41, *p* < 0.05); Negative affect (*R*^2^ = 0.05, *b* = 0.33, *p* < 0.01); Media pressure (*R^2^* = 0.05, *b* = 0.79, *p* < 0.01)	Participants that presented high or medium levels of the moderating variables were likely to present eating pathology symptoms
Salafia and Lemer (2012) United States	136	13.8 (0.88) 12–15	52.9	AMSI BDS	-DEBQ -Bulimia Subscale (EDI)	SEM	Female models: -Performance Stress (*β* = 0.23, *p* < 0.05) - > Body Dissatisfaction (*β* = 0.40, *p* < 0.05) - > Dieting Behaviors (*β* = 0.26, *p* < 0.05) - > Bulimic Symptoms -Relationship Stress (*β* = 0.25, *p* < 0.05) - > Body Dissatisfaction (*β* = 0.40, *p* < 0.05) - > Dieting Behaviors (*β* = 0.26, *p* < 0.05) - > Bulimic Symptoms -Family Stress (*β* = 0.27, *p* < 0.05) -Body Dissatisfaction (*β* = 0.41, *p* < 0.05) -Dieting Behaviors (*β* = 0.26, *p* < 0.05) -Bulimic Symptoms Male Models: -Performance Stress (*β* = 0.30, *p* < 0.05) - > Body Dissatisfaction (*β* = 0.55, *p* < 0.05) - > Dieting Behaviors -Relationship Stress (*β* = 0.37, *p* < 0.05) - > Body Dissatisfaction (*β* = 0.55, *p* < 0.05) - > Dieting Behaviors -Education Stress (*β* = 0.33, *p* < 0.05) - > Body Dissatisfaction (*β* = 0.55, *p* < 0.05) - > Dieting Behaviors -Financial Stress (*β* = 0.30, *p* < 0.05) - > Body Dissatisfaction (*β* = 0.55, *p* < 0.05) - > Dieting Behaviors -Family Stress (*β* = 0.33, *p* < 0.05) - > Body Dissatisfaction (*β* = 0.55, *p* < 0.05) - > Dieting Behaviors	For girls, performance, relationships, and family stress drive the process to dieting behaviors and bulimic symptoms in the end. For boys, all kinds of stress were associated with dieting processes, through body dissatisfaction, however there were no significant associations with bulimic symptoms
Sepúlveda et al. (2021) Spain	180	14.8 (1.5) 12–17	100	BSQ CDI STAIC LOI-CV CAPS FACES-II FQ	EDI-II	Conditional logistic regressions	Eating Disorder group reported higher scores than control groups in: -Drive for thinness (OR = 16.17, 95%CI = 2.78–94.06, *p* < 0.01) -Anxiety state (OR = 5.07, 95%CI = 1.54–16.64, *p* < 0.01) -Obsessive Symptoms (OR = 2.34, 95%CI = 0.90–6.11, *p* < 0.10) -Self-Oriented Perfectionism (OR = 5.03, 95%CI = 1.72–14.69, *p* < 0.01) -Father’s overinvolvement (OR = 7.94, 95%CI = 2.72–23.19, *p* < 0.01) -Mother’s overinvolvement (OR = 5.52, 95%CI = 1.96–15.54, *p* < 0.01) -Mother’s anxiety-state (OR = 6.09, 95%CI = 2.12–17.53, *p* < 0.01)	At the final model, self-oriented perfectionism, and family emotional overinvolvement were the most relevant variables to predict eating disorders compared to control groups
Sharpe et al. (2014) United Kingdom	216	13.6 (0.63) 13–16	100	BES DASS-21 FCS MFQ-RA Peer Scale (IPPA) MFQ-FF Friendship Questionnaire	EDE-Q	Hierarchical lineal regression	Eating Pathology was significantly predicted by more conflict with friends (*β* = 0.19, *p* = 0.006), feeling more alienated from friends (*β* = 0.27, *p* < 0.001), perceive friends to be less helpful to them (*β* = −0.21, *p* = 0.002) and provide less self-validation (*β* = −0.23, *p* = 0.001). After controlling the variable depression, only better communication with friends was associated with eating pathology (*β* = 0.20, *p* = 0.002).	Characteristics related with low-quality friendship were associated with more probabilities of presenting disordered eating symptomatology
Shomaker and Furman (2009) United States	199	18 (0.51) 16–19	49,8	PPAQ PSPS SDBPS	EAT-26	SEM	Following the full model (*X*^2^_*(df* = 123)_ = 216.1, *p* = 0.001; CFI = 0.91; RMSEA = 0.06). Interpersonal pressure to be thin (*b =* 0.27, *p* < 0.001), interpersonal criticism (*b =* 0.16, *p* < 0.05) and disordered eating (*b =* 0.63, *p* < 0.001) at time 1 were significantly associated with disordered eating at time 2 Interpersonal pressure to be thin from mothers (*b =* 0.17, *p* = 0.01), close friends (*b =* 0.16, *p* = 0.02) and romantic partners (*b =* 0.14, *p* = 0.05) at time 1 predicted disordered eating at time 2	These results suggest that interpersonal criticism and interpersonal pressure to be thin provided by mothers, friends, and romantic partners are associated with the onset of disordered eating.
Shroff and Thompson (2006) United States	344	14.6 (1.0) 14–17	100	PFPWDS ACF SSS PAS FSIS PTSF FADS RSES SIAQ	Bulimia Scale of EDI	Multiple regression	Following the full model (*F* = 14.12; *R^2^* = 0.24). Predictors: Composite peer influence (this variable is a combination of the peer influence measures) (*β* = 0.21, *p* < 0.01), Peer Suppression of feelings (*β* = 0.36, *p* < 0.001)	Peer preoccupation with weight/diet, conversations about appearance, peer ideas about perfect body/weight-loss strategies, experienced weight teasing, and self-judgment by external standards. Putting external needs before one’s self or inhibiting one’s self-expression, presented an association with the risk of suffering from bulimia.
Fortes et al. (2015) Brazil	371	13.0 (1.6) 12–16	100	BSQ MPS BRUMS	EAT-26	Multiple linear regression	For the subscale Diet. Predictors: Body Dissatisfaction (*R*^2^ = 0.64, *F*_(1, 370)_ = 119.05, *p* = 0.001). For the subscale Bulimia and Concern about Food. Predictors: Body dissatisfaction (*R*^2^ = 0.10, *F*_(1, 370)_ = 45.98, *p* = 0.001); Perfectionism (*R*^2^ = 0.008, *F*_(2, 369)_ = 5.32, *p* = 0.001); General Mood (*R*^2^ = 0.04, *F*_(3, 368)_ = 5.07, *p* = 0.001) For the subscale Oral Self-Control. Predictors: Body Dissatisfaction (*R*^2^ = 0.10, *F*_(1, 370)_ = 7.57, *p* = 0.007); General Mood (*R*^2^ = 0.04, *F*_(3, 368)_ = 2.33, *p* = 0.05).	Mainly, body dissatisfaction explained the variance in disordered eating, across the different subscales. However, perfectionism and mood state also presented significant associations.
Fortes et al. (2016) Brazil	1,358	13.9(1.0) 12–15	100	RSES SATAQ-3 MPS MDI BRUMS BSQ	EAT-26	SEM	General Model explains 76% variance. The results indicated that Body Dissatisfaction mediates the relationship between media pressure (*b* = 0.36, *p* < 0.05), self-esteem (*b* = 0.14, *p* < 0.05) and mood disturbance (*b* = 0.09, *p* < 0.05) with disordered eating behavior (*b* = 0.62, *p* < 0.01). Media pressure (*b* = 0.27, *p* < 0.01) and depressive symptoms. (*b* = 0.11, *p* < 0.05) also showed a direct relationship with disordered eating behaviors.	All the exposure variables, except for perfectionism, were related to the onset of disordered eating behaviors. Body dissatisfaction acted as a mediator between exposure variables and disordered eating behaviors.
Teixeira et al. (2016) Portugal	575	15.8 (1.6) 11–18	100	CAPS CDFRS RSES	ChEAT	Multiple hierarchical regression and mediation analysis	Following the full model (*R*^2^ = 0.20; *F*_(5, 539)_ = 26.69, *p* < 0.001). Predictors: Body Dissatisfaction (*β* = −0.33, *p* < 0.001), Self-Oriented Perfectionism (*β* = 0.13, *p* = 0.001), Self-Esteem (*β* = −0.21, *p* = 0.001). Mediation analysis revealed that self-oriented perfectionism mediated the association between body dissatisfaction and ChEAT score (95%CI -4.5915 to −0.3610).	Dysfunctional eating behaviors showed a strong association with the presence of self-esteem, self-oriented perfectionism, and body dissatisfaction.
Unikel et al. (2013) Mexico	2,357	16.2 (1.0) 15–19	100	FMRS MCS SRS IBATI SES CES-D	BQREB	SEM	Following the full model (*X*^2^_*(df* = 32)_ = 93.55, *p* < 0.05; CFI = 0.975; RMSEA = 0.04). Affection acted through depressive symptoms (*b* = −0.165) and self-esteem (*b* = 0.407) to internalization of body aesthetic thin ideal. Criticism was also related to internalization thin ideal (*b* = 0.226). Finally, internalization of body aesthetic thin ideal primarily explained disordered eating behavior (*b* = 0.536).	The strongest and direct relationship with disordered eating was with internalization of body aesthetic thin ideal. The association between affection and internalization was mediated by self-esteem and depressive symptoms.
Wade et al. (2015) Australia	926	13 (0.75)	100	MPS Ineffectiveness subscale (EDI) Eating disorder risk	EDE-Q	Latent growth curve	Following the full model (*X*^2^_*(df* = 41)_ = 69.6, *p* = 0.004; CFI = 0.96; TLI = 0.96; RMSEA = 0.05). Negative affect (*b =* 0.15, *p* < 0.05), impulsivity (*b =* 0.21, *p* < 0.01) and gender (*b =* −0.40, *p* < 0.01) were significantly associated with ED-Attitudes at baseline. Impulsivity × negative affect was significantly associated with the growth curve of ED-Attitudes (*b =* −0.60, *p* < 0.05) for high negative affect reactivity group.	Mean levels of ineffectiveness over time mediated the relationship between concerns over mistakes perfectionism at baseline and the change in both of our eating disorder risk variables over time. No support was found for a role of personal standards perfectionism in the mediating relationship.
Zamani et al. (2020) Iran	263	15.8 (1.7) 13–18	100	RSES FRS PAQ-A	EAT-26	Multiple regression	Following the full model (*R* = 0.59; *R*^2^ = 0.35; *p* = 0.004). Predictors: Self-esteem (*β =* 0.59, *t* = 11.9, *p* < 0.001)	These results are contradictory with previous literature, where lower self-esteem scores were associated with higher eating disorder behaviors
Zhu et al. (2016) China	2,172	13.1 (0.84) 11–14	56.7	ASLECL YSQ-SF Impulsivity Subscale of NEO-PI-R	DSM-5 (Binge Eating)	Mediation analysis	EMS mediated the association between Life Stress Events and Binge Eating (*b =* 0.12, *p* < 0.001). Impulsivity moderated the relationship between life stress events and EMS (*b =* 0.03, *p* < 0.05). The relationship between life stress events and EMS was positive and significant when levels of impulsivity were high (*b* = 0.69, *t* = 8.38, *p* < 0.001)	These results show that adolescents with more life stress events, more EMS, and higher levels of impulsivity are likely to present binge eating.

ACF, Appearance Conversations with Friends; ACS, Adolescent Coping Scale; AFQ-Y8, Avoidance and Fusion Questionnaire for Youth; AMSI, Adolescent Minor Stress Inventory; ASLECL, The Adolescent Self-Rating Life Events Check List; BAS^1^, Behavioral Activation System; BAS^2^, Body Appreciation Scale; BDI, Beck Depression Inventory; BDS, Body Dissatisfaction Scale; BES, Binge Eating Scale; BESAA, Body-Esteem Scale for Adolescents and Adults; BIS, Behavioral Inhibition system; BITE, Bulimic Investigatory Test Edinburgh; BPS, Body Perception Scale; BQREB, Brief Questionnaire on Risky Eating Behaviors; BRUMS, Brunel Mood Scale; BSI, Body Satisfaction Instrument; BSQ, Body Shape Questionnaire; BULIT-R, Bulimia Test-Revised; CAPS, Child and Adolescent Perfectionism Scale; CDFRS, Contour Drawing Figure Rating Scale; CDI, Child Depression Inventory; CES-D, Depression Scale from Center for Epidemiological Studies; ChEAT, Children Eating Attitudes Test; CIDI, Composite International Diagnostic Interview; COEDS, The College Eating Disorders Screen; DAPCS, Dependency-oriented and Achievement-oriented Parental Psychological Control Scale; DASS, Depression, Anxiety and Stress Scale; DCS, Diet Competitiveness Scale; DEBQ, The Dutch Eating Behavior Questionnaire; EAT-26, Eating Attitude Test − 26; EBP, Emotional Behavioral Problems; EDBQ, Eating Disorder Belief Questionnaire; EDDS, Eating Disorder Diagnostic Scale; EDEQ, Eating Disorders Examination Questionnaire; EDI, Eating Disorders Inventory; EDRC, Eating Disorder Risk Composite Scale; EI-7, Eysenck Impulsivity Inventory; EMS, Early Maladaptive Schemas; ESS, Experience of Shame Scale; FACES-II, Family Adaptability and Cohesion Scale; FAD, Family Assessment Device; FADS, The Friend Anti-Dieting Scale; FCS, Friends Conflict Scale; FES, Family Environment Scale; FNE, Fear of Negative Evaluation Scale; FQ, Family Questionnaire; FRS, Figure Rating Scale; FMRS, Father and Mother Relationship Scale; FSIS, Friends as a Source of Influence Scale; GHQ, General Health Questionnaire; IBATI, Internalization of body aesthetic thin ideal; IECA, Index of Empathy for Children and Adolescents; IPPA, Inventory of Peer and Parents Attachment; K-GSADS-A, Kutcher Generalized Social Anxiety Scale for Adolescents; KOBI, Quality of Family Interaction Scale; LOI-CV, Leyton Obsessional Inventory Child Version; MBSRQ, Multidimensional Body Self-Relations Questionnaire; MCS, Marshall Criticism Scale; MDI, Major Depression Inventory; MFQ-FF, The McGill Friendship Questionnaire Friend’s Functions; MFQ-RA, The McGill Friendship Questionnaire Respondent’s Affection; MPS, The Multidimensional Perfectionism Scale; MSPSS, Multidimensional Scale of Perceived Social Support; NEO-PI-R, Revised Neuroticism Extraversion Openness Personality Inventory; OBCS, Objectified Body Consciousness Scale; OBVQ, Olweus Bully/Victim Questionnaire; PANAS-C, Positive and Negative Affect Schedule for Children; PAQ -A, Physical Activity Questionnaire for Adolescent; PAS, Peer Attribution Scale; PBI, Parental Bonding Inventory; PDS, Pubertal Developing Scale; PFPWDS, The Perceived Friend Preoccupation with Weight and Dieting Scale; POTS, Perception of Teasing Scale; PPAQ, Pressure to be Physically Attractive Questionnaire; PSPS, Perceived Sociocultural Pressure Scale; PTSF, The Perception of Teasing Scale for Friends; PWT, Peer Weight Teasing; RSES, Rosenberg Self-Esteem Scale; SAAS, Social Appearance Anxiety Scale; SAS-SV, Smartphone Addiction Scale Short Version; SATAQ, Sociocultural Attitudes toward Appearance Questionnaire; SCL-90-R, The Symptom Checklist-90-R; SEM, Structural Equation Modeling; SES, Self-esteem Scale; SIAQ, The Sociocultural Internalization of Appearance Questionnaire; SIBIBCQ, Sociocultural Influences on Body Image and Body Change; SPAS-R, Social Physique Anxiety Scale Revised; SPPA, Self-Perception Profile for Adolescents; SRS, Siblings Relationship Scale; SSQ, Social Support Questionnaire; SSS, The Silencing the Self Scale; STAIC, Staite-Trait Anxiety Inventory for Children; USA, United States of America; WLEIS, Wong and Law Emotional Intelligence Scale; WOCS, Ways of Coping Scale; YSQ-SF, Young Schema Questionnaire Short Form; YSR, Youth Self-Report; ZKPQ, Zuckerman Kuhlman Personality Questionnaire.

### 3.2. Risk of bias assessment

Table 3 presents results of the estimated risk of bias for each study using QUIPS tool. Further analysis provides the frequency of the six assessed domains and (percentages were presented for each label; see Table 4). Most included studies showed low risk for all domains, except for the confounding variables, where the majority presented medium risk. Not all relevant potential variables were considered in the study design or were not reported by the authors.

**Table 3:** Level of risk of bias assessment using QUIPS tool.

Author and year	Study participant	Study attrition	Prognostic factor measurement	Outcome measurement	Study confounding	Statistical analysis and reporting
Altamirano et al. (2011)	Low	Low	Low	Low	Moderate	Low
Argydes et al. (2020)	Low	Low	Low	Low	Low	Low
Bachar et al. (2010)	Low	Low	Moderate	Low	Moderate	Moderate
Bacopoulou et al. (2017)	Moderate	Low	Moderate	Low	Moderate	Low
Baylan et al. (2009)	Low	Low	Low	Low	Low	Low
Beckers et al. (2023)	Low	Low	Low	Low	Low	Low
Boone et al. (2014)	Moderate	Low	Moderate	Moderate	Low	Low
Cella et al. (2021)	Moderate	Low	Low	Low	Low	Low
Ćorić et al., 2023	Low	Low	Low	Low	Low	Low
Costarelli et al. (2011).	Moderate	Low	Low	Low	Moderate	Moderate
Cruz-Sáez et al. (2018)	Low	Low	Low	Moderate	Moderate	Low
Dakanalis et al. (2014)	Low	Low	Low	Low	Low	Low
Evans et al. (2019)	Moderate	Moderate	Low	Low	Moderate	Low
Fortes et al. (2015)	Low	Low	Low	Low	Moderate	Low
Fortes et al. (2016)	Low	Low	Low	Low	Low	Low
Gan et al. (2018)	Low	Low	Low	Low	Moderate	Low
García-Grau et al. (2002)	Low	Low	Low	Low	Moderate	Low
Garrusi et al. (2016)	Low	Low	Moderate	Moderate	Moderate	Moderate
Gomes et al. (2015)	Low	Low	Low	Low	Low	Low
Halliwell and Harvey (2006)	Low	Low	Low	Low	Moderate	Low
Iannaccone et al. (2016)	Low	Low	Low	Low	Moderate	Low
Jones et al. (2020)	Moderate	Moderate	Low	Low	Moderate	Low
Kaewpradub et al. (2017)	Low	Low	Low	Low	Moderate	Low
Kerremans et al. (2010)	Moderate	Moderate	Low	Low	Moderate	Low
Koushiou et al. (2020)	Moderate	Low	Low	Low	Moderate	Moderate
Lee et al. (2018)	Low	Low	Low	Low	Low	Moderate
Lee-Win et al. (2016)	Low	Low	Low	Low	Moderate	Low
Leung et al. (1995)	Moderate	Moderate	Low	Low	Moderate	Low
Li and Li (2021)	Low	Low	Low	Low	Moderate	Low
Macedo-Uchôa et al. (2019)	Low	Low	Moderate	Moderate	Moderate	Moderate
McCabe and Vincent (2003)	Moderate	Low	Low	Moderate	Moderate	Low
Mora et al. (2022)	Low	Low	Low	Low	Low	Low
Pace et al. (2018)	Low	Low	Low	Low	Moderate	Low
Pamies-Aubalat et al. (2022)	Low	Low	Low	Low	Low	Low
Piko et al. (2023)	Low	Low	Low	Low	Low	Low
Rodgers et al. (2014)	Low	Low	Low	Low	Moderate	Low
Rosewall et al. (2018)	Moderate	Low	Low	Low	Moderate	Low
Salafia and Lemer (2012)	Moderate	Moderate	Low	Moderate	High	Low
Sepúlveda et al. (2021)	Low	Moderate	Low	Low	Moderate	Low
Sharpe et al. (2014)	Moderate	Moderate	Moderate	Low	Low	Low
Shomaker and Furman (2009)	Low	Low	Low	Low	Low	Low
Shroff and Thompson (2006)	Moderate	Low	Low	Low	Moderate	Low
Teixeira et al. (2016)	Low	Low	Moderate	Low	Low	Low
Unikel et al. (2013)	Low	Low	Low	Low	Moderate	Low
Wade et al. (2015)	Low	Moderate	Low	Low	Low	Low
Zamani et al. (2020)	Moderate	Moderate	Low	Low	Moderate	Low
Zhu et al. (2016)	Low	Low	Low	Low	Moderate	Low

**Table 4:** Risk of bias summary.

Criterium	Low risk n(%)	Medium risk n(%)	High risk n(%)
Study participant	32(68.1)	15(31.9)	0
Study attrition	38(80.9)	9(19.1)	0
Prognostic factor measurement	40(85.1)	7(14.9)	0
Outcome measurement	41(87.2)	6(12.8)	0
Study confounding	17(36.2)	29(61.7)	1(2.1)
Statistical analysis and reporting	41(87.2)	6(12.8)	0

*n* = number of studies.

### 3.3. Synthesis of primary outcomes

#### 3.3.1. Individual variables

##### 3.3.1.1. Self-esteem

Traditionally, low self-esteem has been associated with a greater likelihood of ED symptoms, particularly in adolescents.

Almost half of the included studies, concretely 24, have analyzed this variable and its relationship with disordered eating. Generally, low self-esteem has been identified as a predictive factor of the onset of EDs symptomatology. However, seven studies did not find significant associations between this variable and negative eating behaviors (Shroff and Thompson, 2006; Baylan et al., 2009; Rodgers et al., 2014; Garrusi et al., 2016; Kaewpradub et al., 2017; Argydes et al., 2020; Pamies-Aubalat et al., 2022).

A total of 12 studies showed self-esteem as a predictive factor with a direct influence on EDs symptomatology. Adolescents with lower self-esteem scores were more likely to develop disordered eating behaviors (McCabe and Vincent, 2003; Altamirano et al., 2011; Gomes et al., 2015; Teixeira et al., 2016; Gan et al., 2018; Rosewall et al., 2018; Jones et al., 2020; Zamani et al., 2020; Cella et al., 2021; Mora et al., 2022; Beckers et al., 2023; Ćorić et al., 2023). In two studies analyzing the role of gender, self-esteem was a strong predictor of the onset EDs symptomatology in both women and men (McCabe and Vincent, 2003; Cella et al., 2021).

Some studies found an interaction between self-esteem and different body attitudes. For example, Cella et al. (2021) observed that negative body feelings and body protection mediate the association between self-esteem and binge eating symptoms for both genders. Two more studies found the same effect regarding body shame (Iannaccone et al., 2016) and body dissatisfaction (Fortes et al., 2016). Thus, presenting negative body attitudes is related with low self-esteem scores and, consequently, a greater presence of disordered eating behaviors during adolescence.

Moreover, self-esteem was also related with other characteristics besides body attitudes. Jones et al. (2020) found that perfectionism was significantly associated with concerns about weight and body through self-esteem. Low scores in this variable were associated with higher scores on perfectionism and more concerns about weight in boys. Also, adolescents who perceived less affection presented lower self-esteem and more disordered eating behaviors (Unikel et al., 2013). Self-esteem also mediates the effect of family functioning and family preoccupation on weight and appearance, therefore participants with low self-esteem were more vulnerable to their family situation, and presented more negative eating behaviors (Leung et al., 1995). The association between self-esteem and EDs symptomatology was also mediated by psychological inflexibility, the adolescents with higher scores in this variable are the ones who presented a lower self-esteem and more likely to present disordered eating behaviors (Koushiou et al., 2020).

##### 3.3.1.2. Body dissatisfaction and attitudes toward body

Body dissatisfaction and attitudes toward the body were also variables traditionally related to the onset of EDs symptomatology. Their relationship with self-esteem and the consequences in the presence of disordered eating behaviors has been analyzed (Fortes et al., 2016; Iannaccone et al., 2016; Cella et al., 2021).

Due to its relevance, 11 articles included body dissatisfaction as a variable of interest. It was observed in 8 studies that body dissatisfaction has been commonly associated with the appearance of EDs symptomatology, and that lower body dissatisfaction was related to a greater presence of disordered eating behaviors for both genders (Altamirano et al., 2011; Boone et al., 2014; Fortes et al., 2015; Garrusi et al., 2016; Teixeira et al., 2016; Cruz-Sáez et al., 2018; Macedo-Uchôa et al., 2019; Argydes et al., 2020; Pamies-Aubalat et al., 2022; Ćorić et al., 2023). This association was also significant in the only study with an entire sample of men (Garrusi et al., 2016).

Boone et al. (2014) also observed interaction effects between body dissatisfaction, personal standards perfectionism, and evaluative concerns perfectionism. Adolescents with higher scores on perfectionism variables presented higher scores in body dissatisfaction and, consequently, more overvaluation of their weight and shape, as well as more bulimic symptoms. The same relationship was found for self-oriented perfectionism in another study (Teixeira et al., 2016). Besides, Salafia and Lemer (2012) showed that high scores on body dissatisfaction were related with higher levels of different kinds of stress for women (family, relationship, and performance stress) and for men (performance, relationship, family, financial and educational stress). These participants were more likely to present dieting behaviors, and for women these behaviors could lead to bulimic symptoms. Cruz-Sáez et al. (2018) observed that the association between body dissatisfaction and disordered eating was mediated by negative self-esteem and negative affect for both genders.

Regarding attitudes toward the body, 7 studies found significant associations between these variables and EDs symptomatology. Negative perception of physical appearance (Costarelli et al., 2011), perception of body size (Gan et al., 2018), social physique anxiety (Gomes et al., 2015) and drive for thinness (Sepúlveda et al., 2021) were directly associated with symptoms of EDs. Argydes et al. (2020) found that overweight preoccupation and body dysphoria were risk factors, while body appreciation was identified as a protective factor for both genders. Li and Li (2021) also showed body esteem as a protective factor against the onset of EDs symptoms. One study showed the influence of media-ideal internalization on body shame and appearance anxiety was mediated by self-objectification for both genders, being the participants with higher scores on these variables and more likely to present dietary restraint or binge eating (Dakanalis et al., 2014). Finally, regular exercise was associated with psychological well-being and with a lower propensity for disordered eating behaviors in adolescents (Gomes et al., 2015).

##### 3.3.1.3. Depression, anxiety, and stress

Because of the higher comorbidity between depression and eating disorders, their relationship has been widely studied. Concretely, 12 articles of this systematic review analyzed this association. Except for two studies (Unikel et al., 2013; Cruz-Sáez et al., 2018), depressive symptoms or negative affect presented a significant and strong association with the appearance of EDs symptomatology for both genders (McCabe and Vincent, 2003; Baylan et al., 2009; Kerremans et al., 2010; Rodgers et al., 2014; Fortes et al., 2015, 2016; Wade et al., 2015; Gan et al., 2018; Rosewall et al., 2018; Evans et al., 2019). Moreover, depressive symptoms lead to an easier internalization of esthetic ideal and developing disordered eating behaviors (Unikel et al., 2013). Previously, it has been analyzed as the mediator role of negative effect between body dissatisfaction and eating behaviors for both genders (Cruz-Sáez et al., 2018).

In the previous section, body dissatisfaction was a mediator between different kinds of stress with diet and bulimic symptoms (Salafia and Lemer, 2012). Zhu et al. (2016) also observed a direct association between life stress events and binge eating. Moreover, this relationship was stronger the higher the levels of impulsivity. Regarding anxiety, 3 studies found an influence of this variable on developing eating disorders for both genders (McCabe and Vincent, 2003; Costarelli et al., 2011; Bacopoulou et al., 2017). Concretely, McCabe and Vincent (2003) observed that anxiety was a strong predictor for bulimic symptoms in men and for extreme weight loss in both women and men.

##### 3.3.1.4. Personal characteristics

High levels of perfectionism have been significantly associated with the onset of EDs symptomatology for both genders (McCabe and Vincent, 2003; Fortes et al., 2015; Teixeira et al., 2016). However, it also presented an indirect relation through self-esteem, mood intolerance (Jones et al., 2020) and body dissatisfaction (Boone et al., 2014). Two studies found a relationship between self-oriented perfectionism and disordered eating for girls (Rosewall et al., 2018; Sepúlveda et al., 2021). Rosewall et al. (2018) also observed an association of socially prescribed perfectionism, proving that social pressure could be a stronger predictor of EDs symptoms in women compared to men.

It has also been observed that other personal characteristics, such as high psychological inflexibility (Koushiou et al., 2020), low emotional intelligence (Li and Li, 2021) and interoceptive awareness (Kerremans et al., 2010) are associated with EDs symptomatology for both genders. Ineffectiveness (McCabe and Vincent, 2003) and covert delinquency (Kerremans et al., 2010) were observed only for men, while inhibited behavior and low effortful control were presented only for women (Kerremans et al., 2010). Obsessive compulsive symptoms were found to be a strong predictor for girls in two studies (Baylan et al., 2009; Sepúlveda et al., 2021). Finally, three studies showed an association between impulsivity and the development of disordered eating behavior during adolescence. In two of the studies, it was observed not only as a significant relationship but also a strong interaction with negative affect, and the negative affect reactivity groups presenting more symptoms of eating disorders for both genders (Wade et al., 2015; Evans et al., 2019). Zhu et al. (2016) showed that the relationship between life stress events and early maladaptive schemas in female and male adolescents was stronger when impulsivity was high, increasing the odds of presenting EDs symptoms.

##### 3.3.1.5. Coping strategies

Two studies focused on analyzing the coping strategies of adolescents and their associations with the appearance of disordered eating behaviors Intropunitive avoidance, avoidance of social support (García-Grau et al., 2002) and escape-avoidance (Lee-Win et al., 2016) showed a direct association with EDs symptoms. Concretely, lifetime prevalence of binge eating was 1.13 times higher with escape-avoidance coping strategy (Lee-Win et al., 2016). These results were supported by Unikel et al. (2013), who included criticism in the analysis and found a positive association with internalization of thin ideal. Finally, two studies found that problem solving could be a protective coping strategy for disordered eating, especially for girls (García-Grau et al., 2002; Baylan et al., 2009).

#### 3.3.2. Sociocultural and social media influence

Eleven articles showed significant associations between the exposure to sociocultural or social media and the onset of EDs symptomatology. Four studies, with 100% women, presented a significant relationship between sociocultural influence (Rodgers et al., 2014), media pressure (Fortes et al., 2016; Rosewall et al., 2018) and internalization of aesthetic thin ideal with disordered eating (Unikel et al., 2013).

Another five studies showed significant associations between media pressure and EDs symptoms according to gender. Argydes et al. (2020) found significant relationships between media pressure and EDs symptomatology in both women and men. Dakanalis et al. (2014) showed the influence of media-ideal internalization on body shame and appearance anxiety mediated by self-objectification for both genders. Two studies (Halliwell and Harvey, 2006; Pamies-Aubalat et al., 2022), found a significant indirect association between weight pressure and body dissatisfaction for both genders, mediated by social comparisons, pressure to lose weight and internalization. Concretely, the odds of presenting disordered eating behaviors increase in participants with higher scores on social comparisons. However, Macedo-Uchôa et al. (2019) only found a significant association between media pressure and EDs symptomatology for men (see Table 2).

Finally, Kaewpradub et al. (2017) conducted a study to analyze the influence of internet and social network use in EDs symptomatology. Moreover, Piko et al. (2023) found that adolescents with smartphone addiction presented more probabilities of developing EDs symptoms. Table 1 showed significant associations between these variables with eating problems, binge eating, purging behavior and taking laxatives.

#### 3.3.3. Family and peers’ influence

Eight studies analyzed the influence of family variables on the appearance of EDs symptomatology.

Some protective factors against the appearance of disordered eating were identified, such as maternal care (Iannaccone et al., 2016), affective involvement (Baylan et al., 2009) and family cohesion (Gan et al., 2018). Specifically, Lee et al. (2018) showed family cohesion to be a positive mediator in the associations between tiredness, boredom, and stress with binge eating (see Table 2).

However, family variables such as paternal achievement oriented psychological control (Pace et al., 2018), parental overinvolvement, mother’s anxiety (Sepúlveda et al., 2021) and family stress (Salafia and Lemer, 2012) presented significant associations with EDs symptomatology. The chances of presenting disordered eating behaviors increase in the presence of these variables. In addition, poor family functioning and family concerns about weight and appearance are related, mediated by self-esteem and body dissatisfaction, with negative eating behaviors in adolescents (Leung et al., 1995; Table 2).

Regarding interpersonal relationships, seven studies identified the influence of these variables on eating behaviors. Perceived peer support was identified as a protective moderator in the relationship between paternal achievement oriented psychological control and EDs symptomatology (Pace et al., 2018; Piko et al., 2023). However, characteristics related with low-quality friendships such as conflicts among friends (Beckers et al., 2023), provide less self-validations, feelings of alienation and perception of less helpful friendships are associated with more probabilities of presenting symptoms of EDs (Sharpe et al., 2014). Moreover, peers’ negative attitudes against body and weight, experienced weight, or appearance teasing (Shomaker and Furman, 2009), and poor communication were more likely to lead to bulimia symptomatology (Shroff and Thompson, 2006).

Finally, being in a romantic adolescent relationship has been identified as another source of pressure and was associated with negative eating behaviors (Shomaker and Furman, 2009; Salafia and Lemer, 2012).

## 4. Discussion

This systematic review aimed to identify the risk factors for EDs symptomatology onset during adolescence, by focusing on both individual and environmental factors.

This study was conducted without limitations for years, in order to provide the most comprehensive overview about EDs risk factors. In fact, the oldest identified article is from almost 30 years ago and was focused on family characteristics and the association with EDs symptoms (Leung et al., 1995). Observations over the past decades, have shown a growing interest in this field, most likely due to the increasing prevalence rates, especially, at early ages (Smink et al., 2012; López, 2017; Galmiche et al., 2019). For that reason, the design of effective prevention and intervention programs has been a priority. However, up to now the proposals in that regard have not shown consistent effective results (Pratt and Woolfenden, 2002; Swanson et al., 2011; Fairburn et al., 2015; Stice et al., 2021).

Consequently, risk factors for EDs should be thoroughly analyzed to identify what is missing from current programs to achieve greater effectiveness, especially in terms of prevention. This systematic review has contributed to fill this gap, as it has identified that, although environmental risk factors (i.e., friends, family and society) were also found to be associated with ED occurrences, prevention programs mainly focus on characteristics like appearance, body weight, and body dissatisfaction, therefore reducing or removing the attention from other relevant areas (Stice et al., 2021). According to this data, the amount of research about the association of peers, family and society characteristics with EDs symptomatology during adolescence are reduced, compared to that of studies about individual characteristics and EDs. For example, from the 47 included studies in this systematic review, only 10 (Halliwell and Harvey, 2006; Unikel et al., 2013; Dakanalis et al., 2014; Rodgers et al., 2014; Fortes et al., 2016; Kaewpradub et al., 2017; Rosewall et al., 2018; Macedo-Uchôa et al., 2019; Argydes et al., 2020; Pamies-Aubalat et al., 2022), eight (Leung et al., 1995; Baylan et al., 2009; Salafia and Lemer, 2012; Iannaccone et al., 2016; Gan et al., 2018; Lee et al., 2018; Pace et al., 2018; Sepúlveda et al., 2021) and six (Shroff and Thompson, 2006; Shomaker and Furman, 2009; Salafia and Lemer, 2012; Sharpe et al., 2014; Pace et al., 2018; Beckers et al., 2023) studied the relationship between society, family and peers with disordered eating behaviors, respectively.

Regarding society, women have always been under more pressure to pursue unrealistic and unattainable appearance ideals. Normally, the standards promoted by current society were based on extreme thinness and looking perfect, and these characteristics were associated with success. The promotion of the thin-ideal and the rejection of other body shapes leaded to a greater body dissatisfaction and the practice of behaviors like dieting, purging or restrictions (Izydorczyk and Sitnik-Warchulska, 2018; Dondzilo et al., 2019). For those reasons, research in this area has focused on women, especially adolescents. In this systematic review, 33 of the included studies had a sample composed entirely or mostly by females. However, in recent years, the investigation with male samples has increased, finding no significant differences compared to women, for the association between sociocultural or media influence with EDs symptomatology (Halliwell and Harvey, 2006; Dakanalis et al., 2014; Kaewpradub et al., 2017; Macedo-Uchôa et al., 2019; Argydes et al., 2020).

In the past decades, communication media was the main way to promote the unrealistic aesthetic ideal (Unikel et al., 2013; Rodgers et al., 2014; Fortes et al., 2016; Rosewall et al., 2018; Macedo-Uchôa et al., 2019; Argydes et al., 2020). However, in recent years, internet and social networking sites have increased and intensified the internalization process of the thin-ideal and social comparisons (Halliwell and Harvey, 2006; Kaewpradub et al., 2017; Pamies-Aubalat et al., 2022). The access to the information and unrealistic aesthetic models are easier and faster, based on an immediate reward system. For example, the use of Instagram in the lockdown was associated with an increase of body dissatisfaction and drive for thinness among young people, who followed more appearance-focused accounts (Vall-Roqué et al., 2021). Therefore, it seems that the education to use social networking sites, personal empowerment, and a correct interpretation of the media information with respect to appearance should be pillars in the EDs prevention programs. The inclusion of these factors could prevent the appearance of body dissatisfaction, body shame or self-objectification symptoms, which are commonly associated with the onset of EDs symptoms (Dakanalis et al., 2014; Saunders et al., 2020).

Considering this information, not only the individual is exposed to the current society, but also their closest and most significant circle, family, and peers. Therefore, the relationship and interactions between the adolescent with their family and peers have been identified as another risk factor of EDs symptomatology (Leung et al., 1995; Shroff and Thompson, 2006; Baylan et al., 2009; Shomaker and Furman, 2009; Salafia and Lemer, 2012; Sharpe et al., 2014; Iannaccone et al., 2016; Gan et al., 2018; Lee et al., 2018; Pace et al., 2018; Sepúlveda et al., 2021). For that reason, family concerns about weight and appearance were associated with features such as low self-esteem (Leung et al., 1995). Also, parental characteristics, like mothers’ anxiety, or parenting styles, such as overinvolvement or psychological control, were associated with more presence of disordered eating behaviors (Salafia and Lemer, 2012; Pace et al., 2018; Sepúlveda et al., 2021). However, there is no agreement about the role of family factors for the onset of EDs symptomatology. Evidence supports that these factors are associated with the exacerbation and maintenance of the symptoms (Sepúlveda et al., 2021).

Furthermore, characteristics like maternal care (Iannaccone et al., 2016), affective involvement (Baylan et al., 2009) and family cohesion (Gan et al., 2018; Lee et al., 2018) were identified as protective factors against disordered eating. It appears that more cohesive families are likely to promote a more stable environment, which in turn is associated with characteristics necessary to deal with the influences of current society, such as higher self-esteem.

However, the influence of peers during adolescence could be just as significant as that of the family. In fact, peer support could be a protective factor of the appearance of EDs symptoms for adolescents whose parents own a controlling parenting style. These individuals perceive the positive reinforcement they need in their equal relationships (Pace et al., 2018; Piko et al., 2023). On the contrary, low-quality relationships based on lack of support, feeling alienated or less self-validation have been identified as risk factors of EDs (Sharpe et al., 2014; Beckers et al., 2023). Moreover, adolescents have been identified as a common audience of appearance-focused accounts on social networking sites (Vall-Roqué et al., 2021) which could lead to exacerbate negative attitudes against weight and body, even suffering from appearance teasing and poor communication (Shroff and Thompson, 2006; Shomaker and Furman, 2009).

In addition to friendship relationships, adolescence is also characterized at the beginning of romantic relationships. This has been identified as another source of pressure, as adolescents desire to be liked by others (Shomaker and Furman, 2009; Salafia and Lemer, 2012). When pursuing to fulfill this need, they may take celebrities as reference. Therefore, they could practice negative behaviors, normally related to EDs symptomatology, with the aim of mirroring famous people who promote unrealistic aesthetic ideals (Unikel et al., 2013; Rodgers et al., 2014; Fortes et al., 2016; Rosewall et al., 2018; Macedo-Uchôa et al., 2019; Argydes et al., 2020). Consequently, the development and the maintenance of EDs symptoms could be reduced if relationships and aesthetic ideals were included in prevention programs, together with training strategies oriented to family members and preparing them to cope with the problem (Moreno-Encinas et al., 2021).

Adolescence is a period characterized by identity formation and emerging independence. Many significant constructs like self-esteem, self-concept, or self-efficacy play an important role in this period. For that reason, it is relevant to develop these characteristics, so they are adaptive and useful for managing stressful events and preventing the onset of psychological problems like EDs (Bardone-Cone et al., 2018). The relevance of individual characteristics in the appearance of EDs symptomatology has promoted a great deal of research in this area. However, this systematic review also showed that the inner circle and the society are key risk factors. For example, if parents were trained in emotion-regulation skills, they could teach these abilities to their children in a more adaptive way. Likewise, if family and friends provide a cohesive, supportive, and validating environment, the likelihood of developing ED symptoms will be reduced. The adolescent is more likely to develop protective personal variables such as strong self-esteem and self-concept. Therefore, the influence of society will have less power, having more skills to manage the pressures to achieve an unrealistic and unhealthy ideal of beauty. If adolescents feel safe in their immediate circle, they will have to make less efforts to try to fit into social standards (Lafrance et al., 2015; Moreno-Encinas et al., 2021).

Regarding individual characteristics, self-esteem has been identified as an essential factor in the appearance of EDs symptoms (Shroff and Thompson, 2006; Baylan et al., 2009; Rodgers et al., 2014; Garrusi et al., 2016; Kaewpradub et al., 2017; Argydes et al., 2020; Mora et al., 2022; Beckers et al., 2023). Therefore, low self-esteem during adolescence showed a significant association with EDs for both genders (McCabe and Vincent, 2003; Altamirano et al., 2011; Gomes et al., 2015; Teixeira et al., 2016; Gan et al., 2018; Rosewall et al., 2018; Jones et al., 2020; Zamani et al., 2020; Cella et al., 2021; Ćorić et al., 2023). However, self-esteem was also a moderator between other characteristics and EDs, especially body dissatisfaction or attitudes toward the body. These characteristics also presented a significant association with EDs (Altamirano et al., 2011; Costarelli et al., 2011; Boone et al., 2014; Fortes et al., 2015; Gomes et al., 2015; Garrusi et al., 2016; Teixeira et al., 2016; Cruz-Sáez et al., 2018; Gan et al., 2018; Macedo-Uchôa et al., 2019; Argydes et al., 2020; Ćorić et al., 2023). This systematic review showed how society could lead to pursue unattainable appearance ideals, failure to achieve these unrealistic goals and the comparisons with models or peers could lead to body dissatisfaction or negative attitudes toward the body (Fortes et al., 2016; Iannaccone et al., 2016; Cella et al., 2021). It has been observed that adolescents with higher self-esteem presented less internalization of the thin-ideal and consequently less probabilities of developing disordered eating behaviors (Bardone-Cone et al., 2018). The presence of low self-esteem and poor family functioning has also influenced the onset of EDs symptoms. Adolescents living in an invalidating environment or being victims of appearance teasing tend to present lower self-esteem and more probabilities of developing negative eating behaviors (Leung et al., 1995; Unikel et al., 2013).

Self-esteem (Jones et al., 2020), body dissatisfaction and attitudes toward body (Boone et al., 2014; Teixeira et al., 2016) were also influenced by perfectionism (McCabe and Vincent, 2003; Fortes et al., 2015; Teixeira et al., 2016), psychological inflexibility (Koushiou et al., 2020) or obsessive-compulsive symptoms (Baylan et al., 2009; Sepúlveda et al., 2021). These associations could be related to establishing unrealistic goals or expectations. Moreover, society, family, and peers could be pressuring the adolescent to pursue those goals (Rosewall et al., 2018). This pressure could lead to obsessive-compulsive behaviors to achieve weight and appearance ideals (Baylan et al., 2009; Sepúlveda et al., 2021). Consequently, body dissatisfaction and negative body attitudes increase as well as the probabilities of developing disordered eating behaviors (Boone et al., 2014; Teixeira et al., 2016; Jones et al., 2020; Koushiou et al., 2020).

However, perfectionism was not the only personal characteristic identified as a risk factor of EDs symptomatology. Ineffectiveness and covert delinquency (McCabe and Vincent, 2003; Kerremans et al., 2010) were associated with disordered eating in men, while inhibited behavior and low effortful control were the features observed in women (Kerremans et al., 2010). Low emotional intelligence was identified as a risk factor for both genders, supporting the individuals with non-adaptive emotion-regulation skills are likely to use strategies like maladaptive eating behaviors to cope with stressful situations (Li and Li, 2021). Impulsivity was analyzed in three studies of this systematic review, being a risk factor for both genders. This trait mediated the relationship between life stress and negative affect with disordered eating, the participants with more symptomatology were those in the negative affect reactivity groups (Wade et al., 2015; Zhu et al., 2016; Evans et al., 2019). In fact, negative affect has been associated with EDs symptoms, playing a relevant role in the internalization of the aesthetic ideal and appearing normally after body dissatisfaction. The dissonance generated between the ideal and the failure to achieve it despite the efforts made leads to not only negative affect (McCabe and Vincent, 2003; Baylan et al., 2009; Kerremans et al., 2010; Rodgers et al., 2014; Fortes et al., 2015, 2016; Wade et al., 2015; Gan et al., 2018; Rosewall et al., 2018; Evans et al., 2019), also high levels of anxiety and stress (McCabe and Vincent, 2003; Costarelli et al., 2011; Salafia and Lemer, 2012; Zhu et al., 2016; Bacopoulou et al., 2017). Therefore, if adolescents are in a period of changing, living in a society focused on thinness and feeling the pressure from their closest circle, they should present active and adaptive coping strategies to face this reality. However, growing in an unsupportive environment which tends to judge appearance leads to develop passive coping strategies, normally related to EDs symptomatology. Intropunitive avoidance, criticism, avoidance of social support and escape-avoidance have been associated with the greater presence of disordered eating behaviors (García-Grau et al., 2002; Unikel et al., 2013; Lee-Win et al., 2016).

This systematic review showed an integrative and comprehensive update on the risk factors that are more likely to lead to EDs symptoms during adolescence. The Supplementary material of this article provides an additional figure to understand the interaction between risk factors. The results presented data from the last 3 decades, from 21 different countries and for both genders, observing non relevant differences by these two variables. Besides, most studies showed a high methodological quality. Although the risk factors for EDs symptomatology have been extensively studied, more research is needed to fully understand the interplay between society, inner circle, and individual characteristics. It has been observed that the etiology of these disorders is complex and involves many factors. However, prevalence rates are still growing, especially at early ages (Smink et al., 2012; López, 2017; Galmiche et al., 2019). Intervention programs have not shown consistent results of long-term effectiveness and up to 80% of people with an ED do not receive an appropriate intervention (Pratt and Woolfenden, 2002; Swanson et al., 2011; Fairburn et al., 2015; Stice et al., 2021). For these reasons, research and constant updating are essential in this area, to identify current gaps and design innovative prevention programs. Knowing the risk factors and the interaction between them, the inclusion of family members in treatments is essential. Training in emotional management and parenting skills is a fundamental point to include in treatments. Furthermore, the integration of these tools in the school dynamics, involving peers and families, could be a measure to take into account from the political sphere. In this way, in addition to information about EDs, practical tools would be provided for adolescents to form their identity in environments where cohesion, support and validation predominate. At the same time, parents could be trained to set limits in a way that is assertive and tolerant, rather than authoritarian and/or overprotective. Moreover, expanding the training offer for healthcare professionals including family and peer therapy would also be a measure to consider. For example, the New Maudsley Method is a novel approach that has demonstrated positive results when administered to parents. This strategy involves training parenting skills in the treatment of disorders. This training method and its extension, including skills for dealing with peers and social pressures, could be a current and innovative solution (Toubøl et al., 2019).

### 4.1. Implications and limitations

Future directions could focus primarily on prevention. With the provision of psychoeducational information by experts in secondary schools, not only for the adolescents but also for educators and families, and the training to promote a safe environment at both school and home, where adolescents can develop and explore their identities and notice the warning signs of the EDs’ onset as soon as possible. The consideration of biological and genetic risk factors involved in EDs could provide a more comprehensive explanation of the onset of these disorders. In this regard, early identification has been associated with a better prognosis (Le Grange and Loeb, 2007). Thus, improvement is needed in intervention programs, especially regarding prevention, which should include in a relevant way the society, family, and peers’ relationships, as well as individual characteristics beyond weight and appearance. Another gap that should be covered is the inclusion of the adolescents’ environments in these programs, to inform about the risks, management of these situations, and effectively prevent them from homes and schools.

Despite its implications and strengths, this systematic review has some limitations. Firstly, although the included studies have analyzed the risk factors for both genders, there are other studies that have only focused on the female population. It’s true that this population has traditionally been more affected by EDs; however, increasing prevalence rates of these problems are also being observed in men. For this reason, further studies involving both genders are required. Secondly, there are more cross-sectional than longitudinal studies included in this review. This type of studies is needed to understand the onset and progression of EDs, as well as testing the long-term effectiveness of prevention and intervention programs. Longitudinal studies require more resources and time, but this type of analysis is also necessary to capture the relative importance that risk factors have at different stages of adolescence. This information could be very useful to identify the risk factors involved in the onset and early stages of EDs. This early identification would allow an earlier and more effective action in terms of prevention and intervention, with the possibility of customize programs to adapt them to specific needs, providing the most appropriate resources in relation to the age of the participant.

The third limitation is that, biological factors were not included, as we focused on psychological factors with the aim to provide information for designing more effective prevention and intervention programs. However, as this may limit the scope of conclusions of this systematic review, future research could include biological factors. Setting of the reviewed studies was another limitation that could bias interpretation and generalization of results: as there were no country restrictions during the search, the included studies are mostly from Europe. However, significant differences were not found in terms of country or culture. Therefore, it seems that beauty ideals are increasingly similar through diverse societies. Since individual risk factors are similar in all cultures, for example, low self-esteem has been seen as a risk factor in most of the articles included. Studies that have analyzed sociocultural, family and peer influences are mostly located in Western societies. However, this only indicates that these variables have been more studied in these places, and that more research is needed into these factors in a wider range of countries. Finally, most of the studies were focused on one area, namely, society, the inner circle, or individual characteristics. To widen the knowledge on the interplay between these three areas in the etiology of EDs, more studies should be carried out in a more integrative and comprehensive way.

To conclude, in future lines of research, the implementation of randomized controlled and longitudinal trials is recommended, specifically, to test and validate the effectiveness of new treatments and prevention programs for EDs. Primarily, these programs should focus on the psychological variables identified as risk factors, rather than aspects related to food or body shape. Furthermore, the role that parents play as a fundamental part of the treatment must be considered; in accordance, parents can not only provide support, but also act as therapists if trained on emotional and parenting skills. Likewise, the inclusion of the management of social pressures, the media and relationships with peers should be relevant elements within treatment and prevention.

## 5. Conclusion

In conclusion, adolescence has traditionally been regarded as a period of changing and identity formation. Adolescents are vulnerable to develop psychological problems if they do not feel they are in a safe environment to define a stable self-concept and self-esteem (Bardone-Cone et al., 2018). This systematic review has shown the importance of including society, family, and peers relationships in interventions and also prevention programs. It is essential that adolescents know the current society and the continuous unrealistic information that people receive about having the perfect body or appearance, and the dangerous behaviors promoted to achieve these goals. It has also been identified that the individual needs to feel affection, support, and cohesion in the family. Moreover, adolescents need to learn how to develop healthy romantic relationships characterized by validation and positive reinforcement. Considering these aspects from an early age, it is likely that the adolescent develops a stronger self-esteem with less possibility of showing high body dissatisfaction. Consequently, negative affect, anxiety, stress, and personal associated traits are likely to result in a more adaptive way, using protective coping strategies such as problem solving. Understanding the etiology of EDs in a comprehensive way could not only have scientific implications but also clinical, for designing innovative and integrative prevention and intervention programs.

## Data availability statement

The original contributions presented in the study are included in the article/Supplementary material, further inquiries can be directed to the corresponding author.

## Ethics statement

Ethical approval was not necessary for the current systematic review as no new participants were recruited for the purpose of the research. However, this study is part of a larger project, approved by the ethical standards of Bioethics Committee of the University Isabel I (Reference: FUI1-014).

## Author contributions

CV, ÁH, MT-S, AJ-G, BM, PR-F, YV-H, and LR-S contributed to design the systematic review. CV designed and conducted the search strategy. CV, ÁH, and MT-S carried out independently the title-abstract and full-text screening. Disparities were solved by discussion. All authors contributed to write the manuscript and approved the submitted version.
