# Supplementary material for: An update on the underlying risk factors of eating disorders onset during adolescence: a systematic review
Source: Front Psychol. 2023 Nov 8;14:1221679. doi: 10.3389/fpsyg.2023.1221679 (PMC10663237; doi:10.3389/fpsyg.2023.1221679)
Supplement: Supplementary file 1 [file Data_Sheet_1.PDF]

Supplementary Material

An Update on the Underlying Risk Factors of Eating Disorders Onset During Adolescence: A Systematic Review

Carmen Varela*, Ángela Hoyo, María Eugenia Tapia-Sanz, Ana Isabel Jiménez-González, Benito Javier Moral, Paula Rodríguez-Fernández, Yadirnaci Vargas-Hernández, Luis Jorge Ruiz-Sánchez

*** Correspondence:** Corresponding Author: carmenvarela@ub.edu

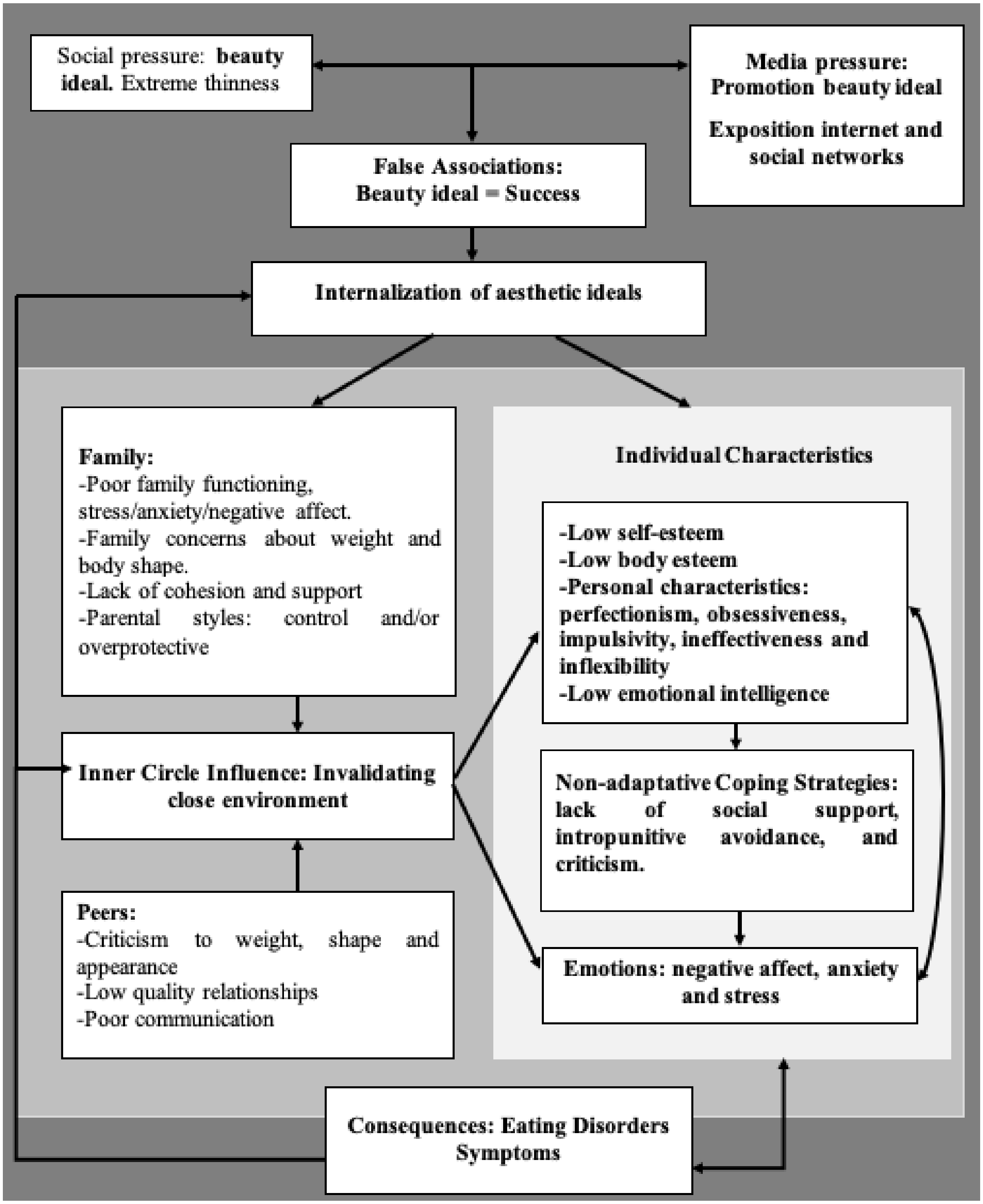

Supplementary Figure 1. Interplay of risk factors
